# Human Action Recognition in Smart Living Services and Applications: Context Awareness, Data Availability, Personalization, and Privacy

**DOI:** 10.3390/s23136040

**Published:** 2023-06-29

**Authors:** Giovanni Diraco, Gabriele Rescio, Andrea Caroppo, Andrea Manni, Alessandro Leone

**Affiliations:** National Research Council of Italy, Institute for Microelectronics and Microsystems, 73100 Lecce, Italy; andrea.caroppo@cnr.it (A.C.); andrea.manni@imm.cnr.it (A.M.); alessandro.leone@cnr.it (A.L.)

**Keywords:** review, human action recognition, smart living, services, applications, context awareness, data availability, personalization, privacy, sensing technology, machine learning, deep learning, signal processing, smart home, smart environment, smart city, smart community, ambient assisted living

## Abstract

Smart living, an increasingly prominent concept, entails incorporating sophisticated technologies in homes and urban environments to elevate the quality of life for citizens. A critical success factor for smart living services and applications, from energy management to healthcare and transportation, is the efficacy of human action recognition (HAR). HAR, rooted in computer vision, seeks to identify human actions and activities using visual data and various sensor modalities. This paper extensively reviews the literature on HAR in smart living services and applications, amalgamating key contributions and challenges while providing insights into future research directions. The review delves into the essential aspects of smart living, the state of the art in HAR, and the potential societal implications of this technology. Moreover, the paper meticulously examines the primary application sectors in smart living that stand to gain from HAR, such as smart homes, smart healthcare, and smart cities. By underscoring the significance of the four dimensions of context awareness, data availability, personalization, and privacy in HAR, this paper offers a comprehensive resource for researchers and practitioners striving to advance smart living services and applications. The methodology for this literature review involved conducting targeted Scopus queries to ensure a comprehensive coverage of relevant publications in the field. Efforts have been made to thoroughly evaluate the existing literature, identify research gaps, and propose future research directions. The comparative advantages of this review lie in its comprehensive coverage of the dimensions essential for smart living services and applications, addressing the limitations of previous reviews and offering valuable insights for researchers and practitioners in the field.

## 1. Introduction

Smart living is an innovative lifestyle that leverages technology to improve quality of life, increase efficiency, and minimize waste. This concept is widely studied by scholars and researchers, who emphasize its various dimensions such as technology, security, health, and education [1]. The smart living lifestyle is predicated on the integration of advanced information and communication technology (ICT), smart sensing technology, ubiquitous computing, big data analytics, and intelligent decision-making to achieve efficient energy consumption, better healthcare, and a general improvement of the services offered to the society towards a high standard of living [2,3].

From a more general perspective, smart living is closely related to the concept of smart cities, which seeks to enhance citizenship characteristics such as awareness, independence, and participation [4]. It aims to transform life and work through ICT, promoting sustainable economic growth and high quality of life while preserving natural resources through participatory governance [5]. Central to this concept is creating benefits for citizens, considering their welfare and participation [6]. Smart living technologies empower users to access and analyze information related to their lives, including personal health and living conditions [3]. As proposed by Giffinger et al. [4], a smart city framework encompasses six main components: smart economy, smart people, smart governance, smart mobility, smart environment, and smart living. The integration of stakeholders such as people, machines, devices, and the environment is crucial for the realization of smart living, which includes aspects such as smart lighting, smart water, smart traffic, smart parking, smart buildings, smart industry, location/context-based services, and many others [7].

Although smart living is driven by intelligent networking and immersive information, it is essential to emphasize the quality of living facilitated by smart technology under sustainable conditions rather than solely driven by technological innovation [8]. As the definitions of smart living continue to evolve with advancements in real-time monitoring systems, it is essential to adapt smart designs and accommodate smart devices, intelligent technology, and sensors to foster a more sustainable and efficient lifestyle for individuals and communities [7,9]. In such a technological landscape, HAR is an integral component of smart living, contributing significantly to relevant applications, including home automation, healthcare, safety, and security. In fact, by accurately identifying and interpreting human actions, smart living systems can deliver real-time responses, offering support and assistance tailored to individual needs. Recognizing human actions is paramount for effectively implementing any smart living application, making it a critical area of research and development in pursuing enhanced quality of life and more efficient, sustainable living environments. From a strictly technological perspective, context awareness, data availability, personalization, and privacy are vital dimensions interwoven with HAR in smart living services and applications. Actually, these dimensions are instrumental in tailoring smart living systems to better cater to individual needs and preferences while preserving privacy and ensuring the availability of relevant data.

A cornerstone of effective HAR in smart living services and applications is context awareness, which involves the intelligent perception and interpretation of surrounding environments and situations [10]. By comprehending the context in which human activities occur, smart living systems can respond more appropriately and adapt to specific circumstances. Furthermore, adaptation is intrinsically linked to personalization, allowing systems to deliver customized experiences and services that cater to each user’s unique preferences and requirements [11]. Personalization and context awareness work in tandem to create a seamless, intuitive, and user-centric environment that enhances the overall quality of life [12].

However, implementing context awareness and personalization necessitates collecting, processing, and storing vast amounts of personal data, raising privacy concerns. As smart living services and applications become increasingly intertwined with users’ daily lives, protecting sensitive information and maintaining user trust is paramount [13]. Thus, balancing harnessing data for personalization and preserving privacy is essential. To achieve this equilibrium, advanced privacy-preserving techniques, such as encryption and anonymization, must ensure that user data remains confidential [14]. Lastly, data availability plays a crucial role in the effective functioning of HAR in smart living services and applications. The accessibility and reliability of data are integral to the performance of these systems, as they rely on the continuous flow of information to make informed decisions and deliver personalized experiences. Ensuring data availability is particularly challenging due to the dynamic nature of smart living environments and the necessity to maintain data consistency across various platforms and devices. Developing robust data management strategies and infrastructure is critical to successfully implementing HAR in smart livingsmart living services and applications [15].

This review concentrates on HAR in smart living services and applications by examining the contemporary state of the art through the lens of the dimensions mentioned above: context awareness, data availability, personalization, and privacy. This analysis aims to provide a comprehensive understanding of the current landscape and identify opportunities for further research and development in HAR for smart living services and applications by investigating the existing literature, advancements, and trends. By focusing on these dimensions, this review seeks to elucidate the challenges and potential solutions associated with effectively implementing HAR systems in many smart living environments, ultimately fostering enhanced quality of life and more efficient, sustainable living conditions.

**Figure 1:**
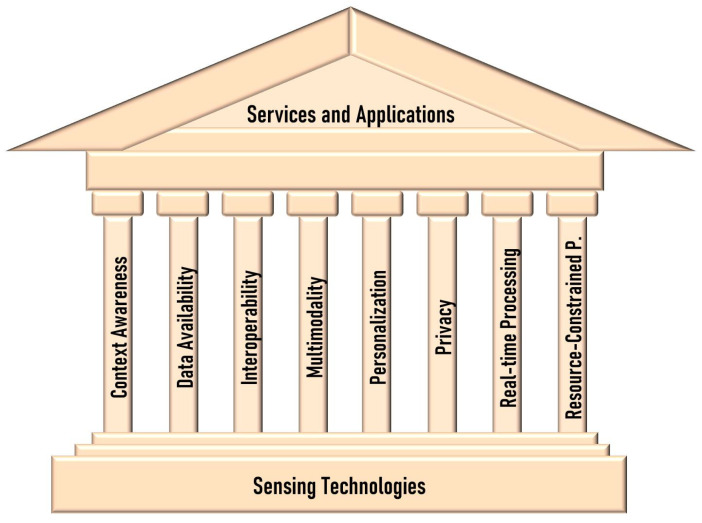
The Temple of Smart Living.

In the previous authors’ work [16], the dimensions of multimodality, real-time processing, interoperability, and resource-constrained processing were analyzed from the perspective of sensing technologies. Composing the dimensions addressed in this review with those previously analyzed outlines what one can define as the temple of smart living. Services and applications form the roof, sensor technologies form the floor, and the dimensions mentioned above form the pillars, as depicted in Figure 1.

The temple of smart living represents the culmination of technological advancements, research, and innovation, creating an environment that fosters a higher quality of life, sustainability, and efficiency. At its core, this temple is supported by pillars representing the fundamental dimensions of smart living: context awareness, data availability, interoperability, multimodality, personalization, privacy, real-time processing, and resource-constrained processing. Each pillar contributes to the strength and functionality of the temple, enabling a seamless integration of services, applications, and sensor technologies. This harmonious combination empowers individuals and communities to lead smarter, more connected lives where technology is harnessed to optimize every aspect of daily living.

As we delve into the analysis of the dimensions of context awareness, data availability, personalization, and privacy in the context of HAR for smart living services and applications, we will explore how these pillars interact and intertwine, forming the foundation upon which this temple stands. By examining the current state of the art and identifying potential areas for further research and development, we aim to unlock the full potential of HAR systems within the realm of smart living. Through this endeavor, we strive to create a future where technology seamlessly integrates with our lives, enhancing our well-being and paving the way for a sustainable and efficient society.

It is important to highlight that this review comprehensively examines the dimensions of HAR within the context of smart living services and applications, specifically emphasizing the utilization of sensors for data collection and analysis. It discusses using sensor modalities and data from various sensing technologies, such as wearables, cameras, ambient sensors, WiFi, and radar-based sensors, to capture human actions and behaviors. By addressing the challenges, opportunities, and current advancements in HAR, particularly concerning context awareness, data availability, personalization, and privacy, the objective of this review is to make a valuable contribution toward the advancement of sensor technologies and their applications in a range of real-world scenarios, including smart homes, healthcare, and smart cities.

### 1.1. Background on HAR in General

HAR is an area of research that focuses on identifying and understanding human activities through the analysis of data acquired from various sensors.

It has applications in many fields such as intelligent video surveillance [17], customer attributes, shopping behavior analysis [18], healthcare [19], military [20], and security [21]. Despite its potential, HAR remains a challenging task due to cluttered backgrounds, occlusions, viewpoint variations, and data noise and artifacts. The recognition of human activities can be approached in two primary ways: using environmental (or ambient) sensors and wearable sensors [22]. Environmental or ambient sensors are fixed at predetermined points, while wearable sensors are attached to the user. In the case of environmental/ambient sensing, smart homes and camera-based systems are examples of HAR. However, these systems face issues such as privacy, pervasiveness, and complexity [23,24].

Deep learning (DL) models, such as convolutional neural networks (CNNs), have been shown to yield competitive performance in visual object recognition, human action recognition, natural language processing, audio classification, and other tasks [25]. CNNs are a type of deep model that learns a hierarchy of features by building high-level features from low-level ones. They have been primarily applied on 2D images, but researchers have started exploring their use for HAR in videos [26].

HAR systems require two main stages: training and testing (evaluation). The training stage involves collecting time-series data of measured attributes from individuals performing each activity, splitting the time series into time windows, applying feature extraction, and generating an activity recognition model using learning methods. During the testing stage, data is collected during a time window, feature extraction is performed, and the trained learning model is used to generate a predicted activity label. There are several design issues in HAR systems, including the selection of attributes and sensors, obtrusiveness, data collection protocol, recognition performance, energy consumption, processing, and flexibility [22,23]. Addressing these issues is crucial for the successful implementation of HAR systems in various real-life applications.

The state-of-the-art HAR systems can be categorized into different groups based on their learning approach, response time, and the nature of the sensors used. Systems can be classified as supervised, semi-supervised, online (often referred also as real-time), offline, and hybrid (combining environmental and wearable sensors). Each of these groups has its own unique challenges and purposes, and they should be evaluated separately.

Hence, HAR is a rapidly evolving field, driven by advancements in DL models, sensor technology, and data processing techniques. The application of CNNs and other DL models to HAR in videos is a promising direction that can potentially improve the performance and capabilities of HAR systems. However, addressing the various design issues and evaluating the performance of HAR systems under realistic conditions remain critical challenges that need to be overcome to fully harness the potential of HAR in various domains.

### 1.2. Background on HAR in Smart Living Services and Applications

In the context of smart living, HAR refers to identifying and analyzing human activities and behaviors using various sensors and computing technologies to provide intelligent, responsive, and personalized services within living environments [27]. These environments include homes, offices, healthcare facilities, and public spaces. HAR-based applications in smart living aim to enhance occupants’ quality of life, safety, and well-being by leveraging technology to automate and adapt to the needs of individuals. The employment of HAR in smart living has led to a wide array of practical use cases. In elderly care, for example, HAR systems can be used to monitor daily activities, detect falls, and assess senior citizens’ health status, enabling timely assistance and improving their quality of life [28]. In smart homes, HAR can facilitate the automation of appliances and lighting based on occupants’ activities and contribute to energy conservation [29]. In security, HAR can detect and alert occupants of potential intruders or suspicious activities [30].

Building on the foundations of HAR in smart living, several critical dimensions play a significant role in ensuring that these systems are truly effective, adaptable, and user-centric. These dimensions include context awareness, data availability, personalization, and privacy, all of which contribute to the overall functionality and success of the smart living experience. Context awareness enables HAR systems to respond intelligently to the varying needs and preferences of occupants in diverse living environments. By incorporating this dimension, smart living solutions can better tailor their services, adapting to different situations and ensuring seamless integration into the daily lives of individuals [31]. On the other hand, personalization empowers users by providing services specifically customized to their needs and preferences, providing a more comfortable, convenient, and intuitive living environment, ultimately enhancing the quality of life for all occupants [32].

Privacy is a vital aspect of smart living, as it helps establish trust and acceptance among users [33]. Respecting occupants’ privacy by safeguarding their data and ensuring transparency in data collection practices can significantly impact the adoption and success of HAR systems in various living environments. Privacy concerns and ethical considerations are important when implementing HAR systems in smart living environments. Ensuring the proper anonymization of data, gaining consent from occupants, and providing transparency in data collection and usage is essential for maintaining trust and user acceptance. Finally, data availability is a crucial dimension that ensures the smooth functioning of HAR systems by providing access to the necessary information for real-time decision-making and analysis [34]. A robust data infrastructure enables smart living solutions to function effectively, adapt to changing circumstances, and deliver a truly intelligent and responsive experience. By incorporating these dimensions into smart living solutions, we can create an ecosystem where HAR-based applications work in harmony with the needs and preferences of occupants, ultimately resulting in a more efficient, secure, and personalized living environment.

#### A Short Note on the Mining of Action

In academic literature, the meaning of the term “action” may vary depending on the context and the authors’ perspective. Some scholars employ the terms “action” and “activity” interchangeably, treating them as synonymous, while others make distinctions between the two.

For a subset of authors, a more structured meaning is assigned to the term “activity” than “action”. For instance, they consider the activity of cooking as a complex process consisting of a sequence of more basic actions. Such actions include pouring water into a container, turning on the stove, waiting for the water to boil, and pouring the hot water into a cup. In this view, activities are perceived as interconnected actions contributing to a specific goal.

On the other hand, some authors ascribe even more basic meanings to the term “action”. They may classify actions as simple, everyday movements or positions, such as walking, sitting, or lying down. In this perspective, actions are closely related to the concept of static or dynamic postures, representing the various states of an individual’s body during different activities.

In the context of this review paper, the authors have opted to utilize the term “action” with a broader connotation. The chosen definition encompasses a wide spectrum of meanings, ranging from high-level activities, which may be influenced by the context, to more basic static or dynamic postures. This inclusive approach to the term “action” allows for a comprehensive analysis and discussion in the review, incorporating diverse perspectives and interpretations from the academic literature.

This review paper makes several significant contributions to the field of human activity recognition (HAR) within the context of smart living. The key contributions and challenges discussed in this work can be summarized as follows:

- Context Awareness: The paper emphasizes the importance of context awareness in HAR for smart living. It highlights the need for systems to perceive and interpret the surrounding environments to provide appropriate responses. This dimension enables intelligent and personalized experiences, enhancing the quality of life for users.
- Data Availability: The continuous flow of information is crucial for informed decision-making and personalized experiences in smart living. The paper underscores the significance of data availability in HAR systems, ensuring the smooth functioning of the systems. It discusses the requirements for selecting datasets that represent everyday routines and tasks in various settings, considering factors such as data quality, sensor usage, and duration of recorded activities.
- Personalization: Personalization plays a pivotal role in HAR for smart living technologies. The paper highlights the benefits of personalized models, which recognize individual uniqueness and tailor actions accordingly. It presents various approaches to personalization, such as identifying similarities between a target subject and individuals in a training set, personalized models based on CNNs and signal decomposition, and maintaining the ordering of time steps in sensor-based HAR.
- Privacy: Addressing privacy concerns is essential for the successful implementation of HAR systems in smart living. The paper discusses the challenges related to sensor choice, data security, and privacy preservation. It presents privacy-preserving techniques, such as device-free sensing approaches, inaudible frequencies, occlusion of personal data, and diversity-aware activity recognition frameworks based on federated meta-learning architecture.

In addition to these key contributions, the paper also discusses the challenges associated with HAR in smart living. It highlights the need for integration of multiple sensing technologies, federated learning for HAR, human-centered design principles, low-power consumption, multi-resident HAR, and ethical considerations and privacy preservation.

The remaining of the paper is structured as follows: Firstly, we explore existing literature reviews in the field to identify gaps and justify the need for this comprehensive study. Next, we outline the criteria for selecting relevant literature and explain our search process. In Section 3, we provide an overview of commonly utilized publicly available datasets in HAR studies, followed by a discussion on widely employed performance metrics for evaluating recognition performance in Section 4. Section 5 focuses on recent research in HAR within smart living, examining aspects such as context awareness, data availability, personalization, and privacy. In Section 6, we analyze the state-of-the-art literature on smart living applications and services. Section 7 critically discusses potential challenges and concerns, offering valuable insights to both researchers and practitioners. Lastly, in Section 8, we conclude the paper by summarizing our findings and providing closing considerations for future research and development in HAR within smart living services and applications.

## 2. Review of Related Works and Rationale for This Comprehensive Study

Recent progress in DL methods for human activity recognition (HAR) has been surveyed by Sun et al. [22], focusing on single-modality and multimodality methods. The need for large datasets, effective fusion and co-learning strategies, efficient action analysis, and unsupervised learning techniques has been emphasized. Saleem et al. [24] present a comprehensive overview of HAR approaches and trends, proposing a HAR taxonomy and discussing benchmark datasets. They also identify open challenges for future research, including high intraclass variations, interclass similarities, background variations, and multiview challenges.

Challenges and trends in HAR and posture prediction are discussed by Ma et al. [35], highlighting four main challenges: significant intraclass variation and interclass similarity, complex scenarios, long untrimmed sequences, and long-tailed distributions in data. They review various datasets, methods, and algorithms and discuss recent advancements and future research directions. Arshad et al. [23] examine the state of HAR literature since 2018, categorizing existing research and identifying areas for future work, including less explored application domains such as animal activity recognition.

A comprehensive survey of unimodal HAR methods is provided by Singh et al. [36], classifying techniques based on ML concepts and discussing differences between ML and DL approaches. Kong and Fu [37] survey techniques in action recognition and prediction from videos, covering various aspects of existing methods and discussing popular action datasets and future research directions. Gu et al. [38] present a comprehensive survey on recent advances and challenges in HAR using DL, examining various DL models and sensors for HAR and discussing key challenges.

Optimal ML algorithms, techniques, and devices for specific HAR applications are examined by Kulsoom et al. [39], providing a comprehensive survey of HAR. They conclude that DL methods have higher performance and accuracy than traditional ML approaches and highlight future directions, limitations, and opportunities in HAR. Gupta et al. [40] present a comprehensive review of HAR, focusing on acquisition devices, AI, and applications, and propose that the growth in HAR devices is synchronized with the artificial intelligence (AI) framework. They also recommend that researchers expand HAR’s scope in diverse domains and improve human health and well-being.

Bian et al. [41] present an extensive survey on sensing modalities used in HAR tasks, categorizing human activities and sensing techniques and discussing future development trends in HAR-related sensing techniques, such as sensor fusion, smart sensors, and novel sensors. Ige et al. [42] survey wearable sensor-based HAR systems and unsupervised learning, discussing the adoption of unsupervised learning in wearable sensor-based HAR and highlighting future research directions. Najeh et al. [43] explore the challenges and potential solutions in real-time HAR using DL and hardware architectures, analyzing various DL architectures and hardware architectures and suggesting new research directions for improving HAR.

After reviewing the literature, it becomes evident that existing survey and review studies can be broadly categorized into two groups: (1) those providing a comprehensive general overview of the field and (2) those focusing on specific aspects such as ML, DL, sensing, and computer vision. However, it is essential to note that, to the authors’ knowledge, there is a lack of research specifically targeting smart living while thoroughly evaluating the existing literature on crucial dimensions essential for smart living from the perspective of services and applications.

These dimensions are crucial for the effective implementation of HAR systems in providing smart and personalized services in living environments. The authors carried out a comprehensive literature analysis, categorizing relevant papers into themes and exploring the state of the art in HAR for smart living. Thus, this review paper goes beyond previous general overviews and focuses on the dimensions essential for smart living services and applications, aiming to offer a comprehensive perspective on the current state of the field.

For this review, an extensive literature analysis was conducted by investigating 511 documents found through a focused Scopus search. This search was constructed to include many pertinent papers by incorporating specific keywords related to HAR and smart living. The search employed the following structure:

TITLE (action OR activity OR activities) AND TITLE (recognition OR classification OR classifying OR recognize OR classified OR classifier OR detector OR detecting OR discriminating OR discrimination) AND TITLE-ABS-KEY (“smart home” OR “smart building” OR “smart environment” OR “smart space” OR “smart living” OR “smart city” OR “smart cities” OR “assisted living” OR “ambient intelligence” OR “smart ambient”) AND PUBYEAR > 2019.

The query sought articles featuring titles that incorporated terms associated with actions or activities and their identification, categorization, or discovery. Additionally, the exploration was narrowed to articles containing title/abstract keywords connected to a range of smart living scenarios, including smart homes, smart buildings, smart environments, smart cities, and ambient intelligence, among other examples. The query also emphasized publications released in 2020 or later, guaranteeing that the analysis considered the latest developments in the domain. The primary factor for choosing a paper for this review was its relevance to one or more of the aforementioned key aspects of smart living. This strategy facilitated the assembly of an extensive and pertinent collection of literature, laying the groundwork for a well-informed and perceptive assessment of HAR within the sphere of smart living.

The papers obtained from the above query can be further classified based on the specific themes they address. In particular, the following, possibly overlapped, categories emerge:

- Context awareness
- Data availability
- Interoperability
- Machine and deep learning
- Multimodality
- Personalization
- Privacy
- Real-time processing
- Resource-constrained processing
- Sensing technologies
- Services and applications

**Figure 2:**
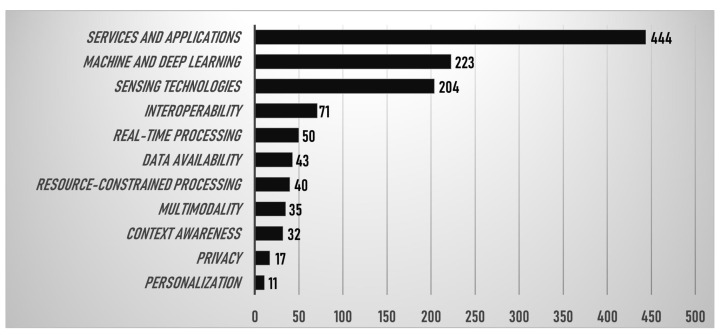
Distribution of research papers on key topics in smart living. Each bar represents the quantity of papers focusing on a specific topic. It is important to note that the quantities of papers may overlap, as individual papers can address multiple topics simultaneously.

The categories listed above with their respective quantities are represented in Figure 2. The plot reveals that the most prominent categories are services and applications, machine and deep learning, and sensing technologies. As said, ML, DL, and sensing have already received extensive coverage in previous review works.

Additionally, interoperability, multimodality, real-time processing, resource-constrained processing, and sensing technologies have been thoroughly analyzed in the previous review study by the authors [16]. Areas such as “personalization” and “privacy” received less attention, indicating potential avenues for future research.

**Figure 3:**
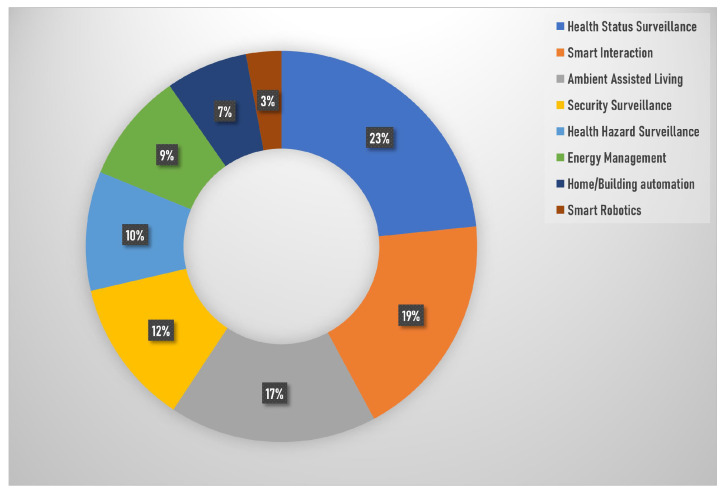
Percentage distribution of services and applications in smart living. The pie plot visually represents the percentage distribution of research papers focusing on specific services and applications within the smart living domain.

This work aims to explore and analyze the concepts of context awareness, data availability, personalization, and privacy, which have not been given much attention in previous reviews. Moreover, the focus of this work is on services and applications that cover various subjects, as shown in Figure 3. These subjects are crucial in creating a seamless and intelligent living environment. Here is a brief overview of these aspects:

- Health Status Surveillance: refers to monitoring and assessing an individual’s health-related aspects such as food intake, lifestyle, well-being, physical activity, sleep, and the use of technology such as robots or mirrors to support healthcare or anomaly detection.
- Smart Interaction: involves various forms of interactive communication between humans and computers, including hand gestures, natural interaction, brain–computer interfaces, and human–computer interaction.
- Ambient Assisted Living (AAL): encompasses technologies and systems designed to support independent living for older adults or individuals with specific needs, focusing on activities of daily living, active and healthy living, as well as assistive and complex human activities.
- Security Surveillance: this relates to using surveillance systems to monitor and detect suspicious or violent activities, ensuring safety and security in various environments.
- Health Hazard Surveillance: involves the monitoring and identifying potential health hazards, such as falls, anomalies, or dangerous situations, particularly in settings such as bathrooms.
- Energy Management: refers to strategies and technologies for efficient energy use, including smart meters, energy-saving techniques, power consumption monitoring, and occupancy-based management.
- Home/Building Automation: involves the automation of various tasks and systems within homes or buildings, utilizing ambient intelligence, intelligent appliances, or white goods (such as household appliances).
- Smart Robotics: the field of robotics encompasses the development and application of robots in various domains or tasks, enhancing automation and intelligent interaction.

As shown in Figure 3, health status surveillance is the most heavily represented area, making up 23% of the total research, followed closely by smart interaction and AAL. This indicates that health-related services and interactive technologies are central to current smart living research. Conversely, areas such as smart robotics and home/building automation make up a smaller portion of the total research, pointing to less explored niches that may have potential for development.

## 3. Common Publicly Available Datasets

Numerous accessible public datasets are often employed for HAR; however, it is vital to understand that these datasets may not fully address specific requirements for smart living services and applications. A key consideration when choosing a dataset for smart living services and applications is the kind of human action incorporated within the dataset. The human actions must be pertinent to the smart living context and represent individuals’ everyday routines and tasks in their homes, workplaces, or urban settings. This way, it is ensured that the HAR models derived from these datasets cater to the distinct demands of smart living solutions.

Another essential factor to consider is the dataset’s subject diversity, including differences in age, gender, and physical capabilities. A broader representation of human activities can be achieved with a diverse group of subjects, which helps create more resilient and versatile HAR models that serve a wider population and can adapt to various individuals and circumstances.

Additional aspects to consider when choosing a dataset for HAR in smart living are data quality, the number of sensors utilized, the placement of these sensors, and the duration of recorded activities. These factors can considerably influence the effectiveness and dependability of HAR models, making it crucial to consider them when selecting the most appropriate dataset for a specific application.

In this review, we have meticulously chosen several pertinent datasets extensively used by the research community for HAR studies. These datasets comprise: Opportunity [44], PAMAP2 [45], CASAS: Aruba [46], CASAS: Cairo [47], CASAS: Kyoto Daily life [48], CASAS: Kyoto Multiresident [49], CASAS: Milan [48], CASAS: Tokyo [50], CASAS: Tulum [48], WISDM [51], ExtraSensory [52], MHEALTH [53], UCF101 [54], HMDB51 [55], NTU RGB+D [56], SmartFABER [57], PAAL ADL Accelerometry [58], Houses: HA, HB, and HC. [59], UCI-HAR [60], Ordonez [61], Utwente [62], IITR-IAR [63].

An overview of the different datasets used by the review works is provided in Table 1.

## 4. Performance Metrics

Classification algorithms are evaluated using various metrics. accuracy (A), recall (R), precision (P), F1-score (F1S), macro-F1-score (mF1S), and specificity (SP) are some commonly used ones. Accuracy (A) is determined by the formula

(1) $$A=\frac{TP+TN}{TP+TN+FP+FN}.$$

This metric measures the ratio of correct predictions made by the model to the total number of predictions made. Recall (R), also known as sensitivity or true positive rate, measures the proportion of relevant instances retrieved. It is determined by the formula

(2) $$R=\frac{TP}{TP+FN}.$$

Precision (P) represents the proportion of true positives among the predicted positives. It is determined by the formula

(3) $$P=\frac{TP}{TP+FP}.$$

The F1-score (F1S) is the harmonic mean of precision and recall, balancing their trade-offs. It is calculated using the formula

(4) $$F1S=2\times \frac{P\times R}{P+R}.$$

The macro-F1-score (mF1S) calculates the average of the F1-scores for each class, treating all classes equally regardless of their size. It is determined using the formula

(5) $$mF1S=\frac{1}{N}\sum_{i=1}^{N}F1S_{i}.$$

Lastly, specificity (SP) is determined by the formula

(6) $$SP=\frac{TN}{TN+FP}$$

and measures the proportion of true negatives among the predicted negatives, reflecting the model’s ability to correctly identify negative instances.

Regarding the symbols above, TP, TN, FP, and FN commonly represent different outcomes in a binary classification problem. They are defined as follows:

- TP: True Positives: the number of positive cases correctly identified by a classifier.
- TN: True Negatives: the number of negative cases correctly identified as negative by a classifier.
- FP: False Positives: the number of negative cases incorrectly identified as positive by a classifier.
- FN: False Negatives: the number of positive cases incorrectly identified as negative by a classifier.

## 5. HAR in Smart Living Services and Applications

The analytical framework presented above provides a comprehensive perspective on the various dimensions of HAR in smart living. However, it is crucial to analyze each dimension critically to ensure that the development of intelligent living environments addresses potential concerns and challenges. Starting from the corpus of papers obtained with the query indicated above, the most representative papers of the dimensions analyzed, namely context awareness, data availability, personalization, and privacy, have been selected. This selection process ensures that the following state-of-the-art examination is based on highly relevant and significant works in HAR.

To facilitate a systematic and organized presentation, each section in the following schematically reports the selected works for each dimension. Dedicated tables provide concise and structured information about each paper, including the methodology employed, the dataset used, the performances achieved, the sensors adopted, and the most prominent actions considered in each work. These tables not only enhance readability but also serve as a quick reference for understanding the critical aspects of each work.

Moreover, the selected papers also focus on identifying and analyzing the actions most considered in the context of HAR in smart living. By prioritizing these actions, researchers can gain insights into the specific human activities that have garnered significant attention in the literature. This approach enables a deeper understanding of the current research landscape and highlights areas that require further investigation and improvement.

By adopting this comprehensive approach, this paper aims to provide a detailed overview of the state of the art in HAR for smart living services and applications. Including the most representative papers and providing essential details about each work’s methodology, dataset, performances, sensors, and prioritized actions contribute to a more thorough understanding of the advancements and challenges in this evolving field.

The papers analyzed in the subsequent sections are carefully compared based on the following evaluation criteria:

- Methods: The methodologies employed in each paper are thoroughly examined and compared. This assessment aims to identify the strengths and weaknesses of different approaches used in HAR for smart living, facilitating a comprehensive understanding of the diverse methods employed in the field. Different methods, such as deep learning models, signal decomposition, or fusion techniques, can have varying impacts on the HAR performance. Understanding the methods employed in the reviewed literature helps researchers and practitioners identify the most effective approaches and explore novel methods.
- Dataset(s): The choice of datasets is significant for evaluating HAR systems. The selected datasets should reflect real-world scenarios, incorporate human actions relevant to smart living, and represent diverse populations. By analyzing the datasets used in previous studies, researchers can identify suitable datasets for their own research and assess the generalizability of existing models.
- Performance: Evaluating the performance of HAR systems is crucial to determine their effectiveness. Performance metrics such as accuracy, recall, precision, F1-score, macro-F1-score, and specificity provide quantitative measures of how well the HAR models perform. These metrics help researchers compare different approaches, identify strengths and weaknesses, and guide the development of more accurate and reliable HAR systems.
- Sensor(s): The choice of sensors used in HAR systems is essential for capturing human actions accurately. Different sensors, such as wearables, cameras, or environmental sensors, can provide distinct types of data and contextual information. Understanding the sensors employed in previous studies helps researchers select appropriate sensors for their own HAR systems and explore sensor fusion techniques to enhance recognition accuracy. This evaluation aimed to assess the types of sensors employed, their placement, and their impact on the performance and practicality of activity recognition in smart living environments. By comparing the sensor configurations, a comprehensive understanding of the advantages and limitations of different setups can be achieved.
- Actions: Recognizing and analyzing specific human actions is the ultimate objective of HAR. By evaluating the actions considered in previous studies, researchers can gain insights into the scope and applicability of HAR in smart living. It helps identify relevant actions for specific domains such as healthcare, security, or energy management, and guides the development of HAR models tailored to specific application sectors. This analysis provides insights into the specific activities that have received significant attention in the literature, facilitating a comprehensive understanding of the research landscape and identifying potential gaps or areas for further exploration.

### 5.1. Context Awareness

Context awareness is a key aspect in designing smart living environments, where systems recognize, interpret, and respond to various contextual factors, including time, location, user preferences, and activities. By understanding and adapting to users’ contexts, these systems can enhance user experience, promote independence, and facilitate convenience [64,65,66,67,68,69]. Some studies have focused on improving feature extraction and resolving activity confusion by using marker-based Stigmergy, a concept derived from social insects that explains their indirect communication and coordination mechanisms (Xu et al. [64]). This approach allows for efficient modeling of daily activities without requiring sophisticated domain knowledge and helps protect the privacy of monitored individuals.

Other research has explored context awareness in HAR for multitenant smart home scenarios, developing methodologies that constrain sensor noise during human activities (Li et al. [65]). By integrating the spatial distance matrix (SDM) with the Contribution Significance Analysis (CSA) method and the broad time-domain CNN algorithm, these approaches ensure accurate and efficient HAR systems. In multi-user spaces, researchers have addressed the challenges of complex sequences of overlapping events by employing transformer-based approaches, such as AR-T (attention-based residual transformer), which extracts long-term event correlations and important events as elements of activity patterns (Kim [66]). This method has shown improved recognition accuracy in real-world testbeds.

Ehatisham-ul-Haq et al. [67] propose a two-stage model for activity recognition in-the-wild (ARW) using portable accelerometer sensors. By incorporating the recognition of human contexts, the model provides a fine-grained representation of daily human activities in natural surroundings. Despite its limitations, the proposed method has achieved reasonable accuracy. Buoncompagni et al. [68] present Arianna+, a framework for designing networks of ontologies that enable smart homes to perform HAR online. This approach focuses on the architectural aspects of accommodating logic-based and data-driven activity models in a context-oriented way, leading to increased intelligibility, reduced computational load, and modularity.

Lastly, Javed et al. [69] explore context awareness in HAR systems for sustainable smart cities. They propose a framework for HAR using raw readings from a combination of fused smartphone sensors, aiming to capitalize on the pervasive nature of smartphones and their embedded sensors to collect context-aware data. The study reports promising results in recognizing human activities compared to similar studies, achieving an accuracy of 99.43% for activity recognition using a deep recurrent neural network (DRNN).

Moreover, context awareness plays a critical role in the development of intelligent, smart living environments, and various research efforts have explored different methodologies and approaches to improve HAR systems by incorporating context-aware information. These advances contribute to the development of sustainable smart cities and healthier societies.

The following presents a detailed analysis of the works described above from the complexity, validity, and generalizability perspective. The purpose is to offer readers a comprehensive understanding of these aspects concerning the proposed solutions for context-aware activity recognition in smart living environments. By examining the complexity of the methods, the validity of their results, and the generalizability of their findings, readers can gain insights into the strengths and limitations of each approach.

Xu et al. [64] propose a novel activity modeling method that utilizes marker-based stigmergy and a directed-weighted network (DwN) with additional context-aware information. The complexity of the method lies in aggregating context information at a low level to generate activity pheromone trails. These trails are represented as a directed-weighted network, capturing individual pheromone sources corresponding to locations. The solution effectively addresses feature extraction and activity ambiguity, achieving good classification performance. The use of stigmergy as context-aware information aggregation enables the modeling of daily activities without requiring sophisticated domain knowledge. The solution also ensures privacy by blurring the individual’s information within the aggregated context-aware data. While the method shows promise, its generalizability might be limited to specific use cases that align with the employed stigmergy paradigm.

Li et al. [65] propose an adaptable methodology for human activity recognition (HAR) in multitenant smart home scenarios, emphasizing context awareness. The complexity of their approach involves constraining sensor noise during human activities by constructing a spatial distance matrix (SDM) based on environmental sensors’ layout. The authors address the challenges of identifying triggers from sensor sequences by considering the coherence characteristics of the home layout and human activities. This approach mitigates behavior confusion errors and improves the overall HAR process. The integration of the SDM with the CSA method and time-domain CNN algorithm ensures accurate and efficient HAR. While the solution demonstrates effectiveness, its generalizability might depend on the specific layout and configuration of the smart home environments.

Kim [66] presents AR-T (activity recognition transformer), a transformer-based approach for real-world activity recognition in multi-user spaces. The complexity lies in extracting long-term event correlations and important events as elements of activity patterns. The solution addresses challenges related to complex sequences of overlapping events triggered by multiple users. The author introduces a duration-incorporated embedding method that allows AR-T to differentiate between events with different durations. The transformer’s ability to compute attention scores and assign importance weights to each context enables effective handling of infrequent but significant contexts. The solution demonstrates improved recognition accuracy compared to existing approaches in real-world testbeds. The generalizability of AR-T is supported by its performance across different datasets, including a seminar room and a smart home testbed, indicating its potential applicability in various multi-user environments.

Ehatisham-ul-Haq et al. [67] propose a two-stage model for activity recognition in-the-wild (ARW) using portable accelerometer sensors. The solution incorporates the recognition of human contexts, providing a fine-grained representation of daily human activities in natural surroundings. The complexity lies in the two-stage supervised classification approach, where primary physical activity of daily living (PADL) is identified in the first stage, and activity-related contexts are inferred in the second stage using accelerometer sensors. The model achieves an average balanced accuracy of 89.43% on the ExtraSensory dataset, demonstrating effectiveness. However, the method’s generalizability might be limited to the specified primary PADLs and context labels used in the experiments, and it may face challenges when handling unforeseen activities and contexts.

Buoncompagni et al. [68] present Arianna+, a framework for designing networks of ontologies to enable smart homes to perform online human activity recognition. The solution focuses on architectural aspects, accommodating logic-based and data-driven activity models in a context-oriented way. The complexity lies in using multiple contexts for encoding knowledge required for activity recognition and classification, which enhances intelligibility, reduces computational load, and enables modularity. The authors demonstrate that the modular network of small ontologies, specialized for each activity, is more intelligible and computationally efficient compared to a single ontology encoding the same knowledge. The solution’s generalizability is supported by its evaluation using the CASAS dataset and its performance comparable to state-of-the-art techniques.

Javed et al. [69] propose a framework for human activity recognition (HAR) in sustainable smart cities, utilizing raw sensor readings from smartphones. The framework leverages a deep recurrent neural network (DRNN) applied to fused smartphone sensor data, including accelerometer, gyroscope, magnetometer, and Google Fit activity tracking module. The complexity lies in capturing context-aware data from smartphones’ embedded sensors and training the DRNN on an extensive dataset. The solution demonstrates promising results with an accuracy of 99.43% for activity recognition. The framework’s generalizability is supported by its potential applications in healthcare, fitness, skill assessment, and personal assistance, contributing to sustainable smart cities.

The works discussed in this section are summarized in Table 2. Table 2 reveals a wealth of information on the strides made in context awareness within the sphere of HAR systems. A broad range of methodologies are utilized in this area, including but not limited to marker-based stigmergy, spatial distance matrix, transformers, machine learning, ontology networks, and deep recurrent neural networks. Furthermore, the researchers have made use of different types of datasets for their studies. These vary from standardized ones such as CASAS and ExtraSensory to datasets that are self-collected, catering to a wide range of activity recognition scenarios.

In terms of performance, the metrics appear to differ from one study to another. However, the overall picture is one of high performance, indicating that the incorporation of context-aware methods into HAR systems is effective. When it comes to data collection, a variety of sensors are utilized, ranging from environmental sensors and smartphone and smartwatch accelerometers, through to more unique sensors such as brightness or seat occupation. This shows that a diverse array of data inputs can contribute to effective context awareness.

Additionally, Table 2 showcases a spectrum of human activities that can be recognized by context-aware systems. These activities range from everyday tasks like cooking, walking, or reading to more complex actions such as moving furniture or engaging in technical discussions. The applications of these context-aware approaches are vast, they cover areas from smart homes and smart cities to individual mobile devices, underlining their versatility and widespread applicability.

### 5.2. Data Availability

Data availability is a major challenge in developing robust HAR systems for smart living services and applications. These systems require accurate and diverse data to learn the intricacies of human movements and interactions, yet acquiring sufficient real-world data for training can be time-consuming and expensive. Moreover, publicly available datasets may not adequately represent the diversity of human actions or the specific contexts in which smart living services and applications operate. To address these limitations, researchers employ various strategies, such as data augmentation, synthetic data generation, simulation, and transfer learning [75,76].

Data augmentation and synthetic data generation techniques help enhance the diversity of training data and mimic real-world situations, enabling models to generalize better and perform well on unseen data.

For instance, Vishwakarma et al. [75] developed SimHumalator, a simulation tool for generating human micro-Doppler radar data in passive WiFi scenarios. Nan and Florea [76] employed data augmentation techniques, such as uniform sampling, random movement, and random rotation, to artificially generate new samples for skeleton-based action recognition.

Annotation of collected data is another critical issue, as it is labor-intensive, time-consuming, and often requires domain experts. To mitigate these annotation difficulties, researchers are exploring the use of unsupervised and semi-supervised learning techniques, which take advantage of the vast amounts of unlabeled data to improve the performance of HAR systems without the need for manual annotation [77,78]. For example, Riboni et al. [77] presented an unsupervised technique for activity recognition in smart homes. Their approach utilized HMMs and employed a knowledge-based strategy incorporating semantic correlations between event types and activity classes. The unsupervised method demonstrated a high level of accuracy, comparable to that of the supervised HMM-based technique reported in existing literature. Additionally, Dhekane et al. [78] proposed a real-time annotation framework for activity recognition, leveraging the change point detection (CPD) methodology.

Semantic and ontology-based approaches can significantly address the data availability problem by facilitating the annotation process, supporting data integration from multiple sources, and enabling reasoning and inference [79]. These approaches can streamline the annotation process by defining a structured and consistent vocabulary for describing human actions and reducing ambiguities and inconsistencies in annotation. Moreover, ontologies can automate certain aspects of the annotation process, reducing the time and effort required by human annotators. By establishing a common semantic framework, researchers can more easily combine and compare datasets, leading to improved performance and generalization in HAR models. Another advantage of semantic and ontology-based approaches is their ability to support reasoning and inference. By capturing relationships and hierarchies between actions, objects, and contexts, these approaches can enable HAR systems to make inferences about unseen or underrepresented actions based on their similarities to known actions [79]. This can fill gaps in the training data and support the development of more robust models even when faced with limited data.

Leveraging ubiquitous sensing devices, such as smartphones, is another way to address data availability. For example, Liaqat et al. [80] proposed an ensemble classification algorithm that uses smartphone data to classify different human activities. This approach significantly expands the potential for widespread adoption of HAR systems, ensuring more people, especially older adults, can benefit from ambient assisted living solutions for improved monitoring and support. Moreover, data availability remains a significant challenge in the development of HAR systems for smart living services and applications. Researchers are adopting various strategies, including data augmentation, synthetic data generation, simulation, transfer learning, unsupervised and semi-supervised learning, semantic and ontology-based approaches, and the use of ubiquitous sensing devices to overcome this challenge. By enhancing the diversity and quality of training data, these approaches help improve the performance and generalization of HAR models.

The following presents a detailed analysis of the works described above from the complexity, validity, and generalizability perspective. The purpose is to offer readers a comprehensive understanding of these aspects concerning the proposed solutions for data availability in smart living HAR. By examining the complexity of the methods, the validity of their results, and the generalizability of their findings, readers can gain insights into the strengths and limitations of each approach.

The first solution, presented by Vishwakarma et al. [75], introduces SimHumalator, an open-source simulation tool for generating human micro-Doppler radar data. The tool uses animation data from motion capture systems and WiFi transmissions to simulate micro-Doppler features that incorporate the diversity of human motion characteristics and sensor parameters. SimHumalator allows users to select target, radar, and signal processing parameters. The generated signatures are experimentally validated using a hardware prototype. The study demonstrates the feasibility of using SimHumalator to generate large human micro-Doppler databases. These synthesized signatures can be used for data augmentation to address the problem of insufficient or unbalanced micro-Doppler training data. The paper presents case studies on using SimHumalator-generated spectrograms for activity recognition applications and showcases classification results from various data augmentation schemes. The complexity of SimHumalator depends on the selected parameters, animation data, and WiFi transmissions. The validity of SimHumalator is supported by the experimental validation of the generated signatures. Generalizability is achieved through the application of the generated data for data augmentation and the demonstration of classification results using the augmented data.

The second solution, proposed by Nan and Florea [76], focuses on skeleton-based action recognition using a neural network approach. The authors utilize a combination of graph convolutional networks (GCN) and temporal convolutional networks (TCN). To address the data availability challenge, the authors employ data augmentation techniques. These techniques involve artificially generating new samples using various transformations applied to the X, Y, and Z coordinates of the skeleton data. The transformations include uniform sampling, random movement, and random rotation. Uniform sampling helps eliminate redundant information and simulate actions collected using sensors with different frame rates. Random movement simulates different positions of the person during data collection, while random rotation simulates different sensor angles. The experiments are performed on the NTU RGB+D dataset, and the proposed model achieves high accuracy and inference speed. The complexity of the data augmentation techniques depends on the number of samples and the chosen transformations. The validity of the approach is demonstrated by the comparative evaluation with state-of-the-art methods and achieving similar or superior performance. The generalizability is supported by the use of a widely used dataset and the model’s ability to perform well on different protocols.

Riboni et al. [77] present an unsupervised technique for multi-resident activity recognition in smart homes. The method leverages unlabeled sensor data stream and ontological reasoning to extract probabilistic associations among sensor events and activities. The authors implemented their algorithms and tested them on a dataset of multi-resident activities performed by couples in an instrumented smart home. The unsupervised approach, combined with hidden Markov models (HMM), achieved high accuracy without the need for labeled datasets. The complexity of the method lies in the unsupervised data acquisition phase and the ontological reasoning process. The validity of the approach is demonstrated by comparing it with a supervised HMM-based method and achieving comparable accuracy. The generalizability is supported by the use of a well-known dataset and the potential for extending the method to handle more residents and collaborative activities in the future.

Dhekane et al. [78] propose a real-time annotation framework for activity recognition based on CPD in the context of smart living. The authors address the challenges of annotating and recognizing activities in streaming, heterogeneous, and noisy smart home sensor data. They propose a similarity-based CPD algorithm that utilizes transfer learning for real-time CPD. The framework comprises four components: feature extraction, classification, data augmentation, and noise handling. The proposed framework achieves state-of-the-art recognition accuracies on various datasets. The complexity of the framework depends on the size and complexity of the sensor data stream, as well as the chosen feature extraction and classification methods. The validity of the framework is supported by achieving high recognition accuracies and outperforming existing methods. The generalizability is demonstrated by the evaluation on multiple datasets and the improvement over the state of the art.

Semantic and ontology-based approaches are highlighted as effective solutions for addressing data availability challenges in HAR systems. They can improve the annotation process by providing a structured and consistent vocabulary for describing human actions and contexts. This streamlines annotation, reduces ambiguities, and automates certain aspects of the annotation process. These approaches also enable data integration from multiple sources by establishing a common semantic framework, facilitating the combination and comparison of datasets collected in different contexts or using different methodologies. Furthermore, semantic and ontology-based approaches can support reasoning and inference, enabling HAR systems to make inferences about unseen or underrepresented actions based on similarities to known actions. This helps fill gaps in training data and improves the robustness of models. The complexity, validity, and generalizability of these approaches depend on the specific semantic and ontology-based techniques employed, as well as the quality and richness of the semantic representations used.

Finally, Liaqat et al. [80] propose an ensemble classification algorithm that utilizes ubiquitous sensing devices like smartphones for multiple activity recognition in older adults. By utilizing smartphone data and employing ML and DL classifiers, the authors achieve high classification accuracy, addressing the data availability challenge by leveraging widely available sensing devices. The complexity, validity, and generalizability of the approach depend on the selected classifiers, the quality of smartphone data, and the performance achieved on diverse datasets. The validity is demonstrated by outperforming other algorithms, while the generalizability is supported by the use of ubiquitous sensing devices that can be widely adopted.

The works discussed in this section are summarized in Table 3. The table highlights the methods used, the types of datasets involved, the performance of the systems, the sensors utilized, and the specific actions the systems can recognize. The strategies range from data augmentation and synthetic data generation to the use of semantic and ontology-based approaches, underscoring the breadth of efforts being made to enhance data diversity and quality.

### 5.3. Personalization

Personalization is crucial in smart living technologies, particularly in the realm of HAR. Recognizing individual uniqueness when performing specific actions can lead to improved recognition accuracy and personalized experiences, overcoming the challenges of a “one-size-fits-all” approach [82]. Researchers have found that by identifying similarities between a target subject and individuals in a training set, emphasizing data from subjects with similar attributes can enhance the overall performance of HAR models [83].

CNNs have been successful in HAR due to their ability to extract features and model complex actions. However, generic models often face performance deterioration when applied to new subjects. Studies have proposed personalized HAR models based on CNN and signal decomposition to address this challenge, achieving better accuracy than state-of-the-art CNN approaches with time-domain features [82]. In healthcare applications, personalization has been explored for classifying normal control individuals and early-stage dementia patients based on activities of daily living (ADLs). Studies have demonstrated that personalized models, considering individual cognitive abilities, exhibit higher accuracy than non-personalized models, underlining the importance of personalization in classifying normal control and early-stage dementia patients [84].

Several studies have proposed novel approaches for sensor-based HAR that focus on personalization by maintaining the ordering of time steps, crucial for accurate and robust HAR systems. They have introduced network architectures combining dilated causal convolution and multi-head self-attention mechanisms, offering a more personalized and efficient solution for sensor-based HAR systems [85]. Researchers have also explored personalized approaches for HAR within smart homes by utilizing a multilayer perceptron (MLP) neural network. The proposed method adapts to individual users’ patterns and habits, achieving high recognition accuracy across all activity classes [86]. Recent work has focused on addressing the challenges associated with recognizing complex human activities using sensor-based HAR.

By exploring hybrid DL models combining convolutional layers with recurrent neural network (RNN)-based models, researchers have demonstrated the potential of these models to contribute significantly to personalization in various applications involving wearable sensor data [87].

To address the challenge of personalization in sensor-based HAR, particularly in healthcare applications, studies have proposed unsupervised domain adaptation approaches that allow sharing and transferring of activity models between heterogeneous datasets without requiring activity labels for the target dataset. This approach enhances the personalization aspect of activity recognition models, allowing adaptation to new, unlabeled datasets from different individuals or settings [88].

Furthermore, personalization plays a vital role in enhancing the effectiveness and efficiency of HAR models. By considering individual uniqueness and utilizing various techniques such as CNNs, RNN-based models, and unsupervised domain adaptation, researchers have made significant strides in creating more tailored and accurate HAR systems for smart living and healthcare applications.

The following presents a detailed analysis of the works described above from the complexity, validity, and generalizability perspective. The purpose is to offer readers a comprehensive understanding of these aspects concerning the proposed solutions for personalization in HAR-based smart living. By examining the complexity of the methods, the validity of their results, and the generalizability of their findings, readers can gain insights into the strengths and limitations of each approach.

The importance of personalized smart living technologies, especially in HAR, is gaining more attention. The uniqueness of individuals and their actions necessitates a more customized approach to improve the effectiveness of the technologies. Harnessing distinctive individual features can significantly enhance recognition accuracy and create personalized experiences, moving beyond a one-size-fits-all approach. This discussion is illustrated in various research, one of which is by Zunino et al., who propose a strategy where data from individuals sharing similarities with the target subject is emphasized, thereby achieving better HAR performance [83]. This process of data selection enhances the model’s understanding of an individual’s action nuances, promoting more precise recognition and a more tailored experience. It allows the model to perform excellently even when training with fewer instances, indicating a high level of efficiency in training complexity and good generalizability.

Gholamiangonabadi et al. propose a personalized HAR model based on CNNs and signal decomposition [82]. This research offers another complexity layer by applying signal processing techniques to extract features from multimodal sensor data, followed by CNN-based classification. The model introduces a personalized touch by selecting the best suited trained CNN version using a portion of the target subject’s data. This method proved superior to other state-of-the-art CNN approaches, suggesting a high level of validity. However, the choice of the best-suited CNN depends on the target subject’s data, which could limit its generalizability to new subjects.

Furthermore, Kwon et al. focus on distinguishing between normal control individuals and early-stage dementia patients based on their ADLs using smart home sensor data [84]. This approach uses a combination of statistical analysis and machine learning techniques such as the random forest classifier (RFC), known for its excellent classification performance [89]. The model’s personalization aspect is seen in the setting of anomaly detection criteria based on cognitive function, which enhances classification accuracy by considering each individual’s unique range of activities. While the RFC’s strength in handling hundreds of independent variables and large amounts of learning data promises good generalizability, the dependency on personalized anomaly detection criteria may introduce complexity during adaptation to new subjects.

Hamad et al. propose a novel network architecture that combines dilated causal convolution and multi-head self-attention mechanisms to address variations and complexities in human behaviors [85]. This architecture maintains the ordering of time steps, crucial for accurate HAR systems. The methodology contrasts with recurrent neural networks (RNNs), which are inherently limited due to their sequential computation. This approach enables effective parallelization of operations, offering efficiency and potential scalability to larger datasets.

In contrast, Gorjani et al. use a multilayer perceptron neural network to recognize different human activities using data from wrist and ankle-worn devices [86]. Their approach, providing a high level of personalization by adapting to individual users’ patterns and habits, reveals high recognition accuracy across all activity classes, suggesting strong validity. However, due to its high level of personalization, its generalizability may be limited.

Mekruksavanich et al.’s research involves the application of RNN-based deep learning models in recognizing complex human activities using sensor-based HAR [87]. Their approach of using hybrid models, combining convolutional layers with RNN-based models, exploits the strengths of CNNs and RNNs for complex activity recognition tasks, thus ensuring high accuracy, validity, and robustness of the model. However, the complexity of the models and their heavy dependence on specific wearable sensor data may affect the generalizability of the approach.

Lastly, Sanabria et al. propose an unsupervised domain adaptation approach (UDAR) for sensor-based HAR, combining knowledge-driven and data-driven methods for feature alignment [88]. This approach allows the model to adapt to the variations in unlabeled datasets, indicating high robustness and adaptability. Although it demonstrates high recognition accuracy and strong robustness against sensor noise, its dependence on feature alignment and ensemble learning may introduce complexities in model development, affecting its scalability.

The works discussed in this section are summarized in Table 4. It is apparent that a key trend in the current research landscape is the development of personalized HAR models which aim to improve the recognition accuracy. This is typically achieved through the innovative use of diverse methodologies including but not limited to signal decomposition, dilated causal convolution, MLP neural networks, and unsupervised domain adaptation.

In terms of datasets, both pre-existing ones like MHEALTH, WISDM, and UCI-HAR, and self-collected datasets are being used. Notably, the trend towards collecting unique datasets for particular studies demonstrates the increasing demand for capturing individual-specific data to boost the personalization of HAR models. When considering performance, the use of personalized approaches generally delivers high accuracy, reaching average accuracy above 90% in several studies, which indicates the efficacy of these methods. It can be discerned that a range of sensors—accelerometers, gyroscopes, magnetometers, and environmental sensors—are being employed in the studies to detect a wide variety of actions. This reveals an interdisciplinary trend where diverse sensor technologies are deployed to cater to the unique requirements of each study.

Lastly, the variety of actions examined in these studies, spanning from everyday activities such as cooking or walking to specific tasks such as gym activities, underlines the broad applicability of HAR systems and the importance of personalization across different contexts.

### 5.4. Privacy

Privacy concerns in HAR have been addressed through two primary aspects: sensor choice and data security. Researchers have focused on exploring sensing modalities that do not capture privacy-sensitive information. Device-free sensing approaches have emerged as a viable alternative to intrusive body-worn or ambient-installed devices, with examples such as WiFi and radar-based sensors [91,92,93,94]. Privacy-preserving techniques have also been developed for traditional audio and video-based methods, using inaudible frequencies or occluding person data [95,96,97]. Studies have demonstrated the importance of contextual information in HAR and its potential for preserving privacy without sacrificing performance [97].

Researchers have also developed privacy-preserving HAR systems using low-resolution infrared array sensors, showcasing promising recognition accuracy [98]. Furthermore, inaudible acoustic frequencies have been explored for daily activity recognition, resulting in privacy-preserving accuracies of up to 91.4% [95]. Data security has been addressed through local training via federated architecture, preventing data from being sent to third parties [99]. Detection of spoofing attacks in video replay and vulnerability to adversarial attacks in video and radar data have also been investigated [100,101].

Diversity-aware activity recognition frameworks based on federated meta-learning architecture have been proposed, which preserve privacy-sensitive information in sensory data and demonstrate competitive performance in multi-individual activity recognition tasks [99]. Studies have also revealed radar-based CNNs’ vulnerability to adversarial attacks and a connection between adversarial optimization and interpretability [100]. Lightweight DL-based algorithms capable of running alongside HAR algorithms have been developed to detect and report cases of video replay spoofing [101].

Researchers have proposed novel methodologies for explainable sensor-based activity recognition in smart-home environments, transforming sensor data into semantic images while preserving privacy [102]. Federated learning has also been leveraged to develop personalized HAR frameworks, allowing training data to remain local and protecting users’ privacy [103]. The studies collectively contribute to addressing privacy concerns and advancing HAR research. In this section, privacy concerns in HAR have been addressed by focusing on sensor choice and data security. Researchers have explored device-free sensing approaches that do not require intrusive sensors and developed privacy-preserving techniques for traditional audio and video-based methods. Contextual information has been shown to play a crucial role in HAR performance and privacy preservation. Data security has been enhanced through federated learning and local training, reducing the need to share data with third parties. Research has also investigated vulnerability to adversarial attacks, spoofing detection, and the connection between adversarial optimization and interpretability. Innovative methodologies have been developed to provide explainable activity recognition while preserving privacy in smart-home environments. Federated learning has been employed to create personalized HAR frameworks that protect users’ privacy. These studies collectively represent the state of the art in addressing privacy concerns in HAR and pave the way for advancements in the field, ensuring that users’ privacy is maintained while delivering reliable recognition performance.

The various solutions reviewed address privacy concerns in HAR primarily through innovative sensing modalities, data security measures, and advanced learning techniques. The fundamental aspects of complexity, validity, and generalizability are crucial for understanding the efficacy and potential implications of these solutions.

Complexity refers to the degree of intricacy of the developed model, the computational resources it requires, and how easily it can be implemented or incorporated into existing systems. Yan et al. [97], for instance, proposed a method involving image segmentation for occluding human target data in privacy-preserving HAR. While the approach is more complex than traditional HAR, it manages to maintain a high level of accuracy by preserving the target’s shape. This model balances the trade-off between privacy protection and performance, but its complexity could potentially limit its real-world implementation. On the other hand, Yin et al. [98] proposed a device-free sensing system using low-resolution infrared array sensors, an approach that is less complex, ensures users’ privacy, and reduces the deployment cost.

Validity refers to the extent to which a model correctly identifies or predicts the phenomenon it is intended to study. In the case of Yan et al. [97], the validity of the proposed model is demonstrated by the high accuracy rates achieved when only contextual information was provided to the network. On the contrary, Yin et al. [98] showed high validity of their approach with an impressive recognition accuracy of 98.287% for typical daily activities, surpassing existing machine learning methods.

Generalizability refers to how well a model or method can be applied to various scenarios or populations. Iravantchi et al. [95] developed a privacy-preserving device using inaudible frequencies for activity recognition, achieving over 95% classification accuracy across all environments, indicating its high generalizability.

Similarly, Shen et al. [99] proposed a diversity-aware activity recognition framework, which demonstrates superior generalization ability compared to other models in multi-individual activity recognition tasks. This high generalizability shows its potential for widespread implementation in multiple contexts.

However, in the case of Huszár et al. [101], their model may not generalize well to other scenarios since it was specifically designed to detect spoofing attacks in video replay for automatic HAR applications. The authors themselves noted the need for fine-tuning of the model for better-fit cases with higher image pixel density.

Arrotta et al. [102] proposed a novel methodology for explainable sensor-based ADL recognition. While their method is relatively complex, involving transformation of sensor data into semantic images and the application of multiple Explainable AI (XAI) methods, it provides highly understandable insights, as validated by a user study. The approach’s generalizability is demonstrated by its application on two datasets, yet it remains to be seen how it performs in a broader range of real-world environments.

On the data security aspect, researchers have proposed solutions such as local training through federated architecture, spoofing attack detection, and adversarial attack vulnerability investigation. The paper by Yu et al. [103] proposed a federated HAR framework that addresses privacy preservation, label scarcity, real-time processing, and heterogeneity patterns in HAR. Despite its complexity, the framework demonstrated its validity with superior performance over existing methods, and its generalisability was demonstrated by conducting experiments on two diverse real-world HAR datasets.

The works discussed in this section are summarized in Table 5. From an examination of Table 5, a number of key insights can be gleaned about current research trends and methods in addressing privacy concerns within HAR.

Researchers are employing a range of innovative methods to balance the need for precise activity recognition with privacy considerations. These methods often involve leveraging advanced technologies such as federated learning, LSTM-CNN, Inflated 3D ConvNet, and device-free sensing modalities such as WiFi and radar-based sensors. A prominent trend is the utilization of less intrusive or privacy-preserving sensors. These include infrared arrays, WiFi signals, and even ultra-wideband radars. This indicates a shift towards non-wearable or non-intrusive sensors that minimize privacy intrusion while still effectively recognizing human activities.

The exploration of federated architectures stands out as a significant trend for ensuring data privacy. Federated learning allows model training to occur locally on devices, preventing sensitive data from being transmitted to third parties. This, along with the use of minimalist data pre-processing and many-objective evolutionary algorithms, suggests a drive towards maximizing privacy without sacrificing model performance.

From a performance perspective, it is evident that these privacy-preserving approaches do not significantly compromise the accuracy of the HAR models. Several studies reported average accuracy rates upwards of 90%, indicating the effectiveness of these methods in a privacy-considerate manner. The range of actions studied, from everyday household activities to more specific tasks, further underscores the comprehensive applicability of these privacy-preserving HAR techniques across various domains.

## 6. Smart Living Services and Applications

HAR systems have shown great potential in enhancing smart living services and applications, spanning diverse areas such as assisted living, health status surveillance, health hazard surveillance, energy management, security surveillance, and natural interaction. These applications aim to improve the lives of seniors, monitor health conditions, optimize energy consumption, and enhance security across various settings by utilizing cutting-edge ML techniques, sensor data, and innovative strategies like radar phase information and WiFi-based recognition [108,109].

To illustrate the practical applications of HAR and provide concrete insights into the challenges related to the dimensions of context awareness, data availability, personalization, and privacy, some examples and case studies are presented. These real-world scenarios shed light on how HAR can be applied in various domains and highlight the specific challenges associated with each dimension.

- **Context Awareness**: Context awareness enables HAR systems to respond intelligently to occupants’ needs and preferences in diverse environments. In the domain of smart homes, context-aware HAR can automatically adjust lighting, temperature, and other environmental settings based on occupants’ activities and preferences. For example, when a person enters a room, the system can detect their presence and adjust the room’s lighting to an appropriate level. Context-aware HAR also finds applications in smart healthcare, where it can monitor and analyze patients’ activities to detect anomalies and alert healthcare providers in case of emergencies.
- **Data Availability**: Data availability in HAR refers to the availability of data that is useful for model training. In order to train effective HAR models, it is essential to have access to real-world data that capture human activities in various contexts. However, collecting and labeling real-world data can present challenges, particularly in cases where the detection of harmful situations such as falls is necessary. To overcome these difficulties, researchers have adopted methods based on simulated, synthetic, and augmented datasets.
- **Personalization**: Personalization plays a key role in enhancing the effectiveness of HAR systems in smart living. In the field of healthcare, personalized HAR models can improve recognition accuracy and provide tailored support for individuals with specific conditions. For example, personalized HAR can be used to classify normal control individuals and early-stage dementia patients based on ADLs, leading to better diagnosis and treatment strategies. In smart homes, personalized HAR models can adapt to individual users’ patterns and habits, allowing for high recognition accuracy across all activity classes.
- **Privacy**: Privacy is a critical consideration in the design and implementation of HAR systems. To address privacy concerns, researchers have explored various techniques. For instance, device-free sensing approaches, such as WiFi and radar-based sensors, have been used to capture activity information without compromising privacy. Privacy-preserving techniques, including inaudible frequencies and occlusion of personal data, have been developed for traditional audio- and video-based methods. Contextual information has been shown to play a vital role in preserving privacy without sacrificing performance.

HAR systems have been particularly effective in assisted living applications, improving the quality of life for elderly individuals and those with chronic conditions, while supporting healthcare professionals and caregivers in providing more effective care [19,108]. For instance, HAR has been used to monitor the daily routines of older persons and detect deviations in their behavior as well as to recognize fall activities and notify caregivers or medical professionals during emergencies [109,110]. HAR systems can also identify ADLs in smart home environments and provide valuable information about older adults’ health conditions to family members, caretakers, or doctors, helping to adapt care plans as needed [19].

Health status surveillance plays a significant role in smart living services and applications, addressing the needs of an aging population and patients with neurodegenerative disorders. ML and signal processing techniques, such as support vector machines (SVMs) and random decision forest classifiers, can be employed to disaggregate domestic energy supplies and assess ADLs [111]. Preventive healthcare can also be supported by recognizing dietary intake using DL models like EfficientDet [112] and monitoring physical activity through smartphone accelerometer sensor data and DL models [113].

Health hazard surveillance is essential for the well-being and safety of elderly populations in smart living services and applications. HAR systems can help monitor older adults’ daily activities, identify potential hazards, and alert caregivers or medical professionals in emergencies. This approach allows for timely intervention and can prevent the exacerbation of health conditions or accidents, ensuring a safer environment for seniors [114,115].

Energy management in smart homes and buildings can be improved by incorporating HAR into smart living services and applications. By understanding and monitoring human behavior, these systems can optimize energy consumption while maintaining comfort for the occupants. HAR can be employed to optimize energy consumption in heating, ventilation, and air conditioning (HVAC) systems [116] and in building energy and comfort management (BECM) systems by learning users’ habits and preferences and predicting their activities and appliance usage sequences [117].

Security surveillance can be significantly enhanced by applying HAR in smart living environments. Accurate identification and classification of activities based on visual or auditory observations can contribute to a safer, more secure environment in various contexts. This can be achieved through approaches like using a fine-tuned YOLO-v4 model for activity detection combined with a 3D-CNN for classification purposes [118] and employing SVM algorithms to classify activities based on features extracted from audio samples [119].

In addition to enhancing security surveillance in various settings, such as video surveillance, healthcare systems, and human-computer interaction, HAR systems can provide accurate activity detection and recognition, offering valuable insights for security personnel in real-world scenarios like university premises or urban environments [118,119]. By incorporating radar phase information and WiFi-based approaches, HAR systems can significantly improve natural interaction in smart living services and applications, providing low-latency, real-time processing, and touch-free sensing benefits for various applications, including elder care, child safety, and smart home monitoring [120,121].

Natural interaction in smart living services and applications can be improved by recognizing human actions and gestures in a non-intrusive, privacy-preserving manner. Exploiting radar phase information and WiFi-based approaches in HAR can enhance natural interaction significantly in smart living services and applications, providing low-latency, real-time processing, and touch-free sensing benefits. Recent advancements in radar phase information extraction from high-resolution range maps (RM) offer a promising alternative to traditional methods, such as micro-Doppler spectrograms, which suffer from time-frequency resolution trade-offs and computational constraints [120]. The histogram of oriented gradients (HOG) algorithm can capture unique shapes and patterns in the wrapped phase domains, demonstrating high classification accuracy of over 92% in datasets of arm gestures and gross-motor activities. By employing various classification algorithms, such as nearest neighbor, linear SVM, and Gaussian SVM, improved performance and robustness in various activity aspects, including the aspect angle and speed of performance, can be achieved [120].

The ubiquity of WiFi devices in modern buildings provides an opportunity for cost-effective, touch-free activity and gesture recognition systems. Human activities and gestures can be accurately recognized by harnessing the channel state information (CSI) value provided by WiFi devices [121]. Median filtering techniques can be applied to filter out noise from the CSI, and massive features can be extracted to represent the intrinsic characteristics of each gesture and activity. Using data classification algorithms, such as random forest classifier (RFC) and SVM with cross-validation techniques, can achieve high recognition accuracy rates of up to 92% and 91%, respectively [121].

Overall, the integration of HAR systems into smart living services and applications offers a promising avenue for enhancing the lives of seniors, monitoring health conditions, optimizing energy consumption, and bolstering security across various settings. With continued advancements in ML, sensor technology, and innovative recognition strategies, the potential of HAR systems in smart living services and applications will undoubtedly continue to grow, paving the way for more sustainable, secure, and supportive living environments.

The works discussed in this section are summarized in Table 6. It is noticeable that a significant portion of the referenced works focuses on assisted living, which underlines the role of HAR in enhancing the quality of life for the elderly and those with chronic conditions through sophisticated monitoring systems. These employ a variety of techniques ranging from neural networks and object detection to more complex methods such as human–object interaction (HOI) detection and scene understanding.

HAR has also demonstrated significant potential in health status and health hazard surveillance, with the studies using techniques such as SVM, RFC, and DT among others for health monitoring and anomaly detection. The application of HAR in preventive healthcare has been seen through the integration of innovative approaches such as deep learning models for dietary intake recognition.

Table 6 further emphasizes the role of HAR in Energy Management, with a study employing a combination of computer vision and ML techniques for efficient energy consumption. The application of HAR in security surveillance is also notable, with methodologies including YOLO-v4 and SVM algorithms being utilized for recognizing and classifying suspicious activities.

Finally, there is an increasing trend towards improving Natural Interaction in smart living environments. These applications exploit radar phase information and WiFi-based approaches to recognize human actions and gestures, contributing to a non-intrusive and privacy-preserving environment. This is facilitated by a variety of methods such as histogram of oriented gradients (HOG), NN, and deep CNNs.

## 7. Discussion: Open Issues and Future Research Directions

The integration of multiple sensing technologies is a promising research direction for improving HAR systems in smart living services and applications. Combining data from various sensor types, such as wearable devices, cameras, and ambient sensors, can yield richer contextual information and lead to more accurate and reliable activity recognition. As each sensor type has its strengths and weaknesses, their integration can compensate for individual limitations and provide a more comprehensive understanding of users’ activities. Future research should explore efficient sensor fusion techniques and investigate how to effectively exploit complementary sensor data for improved activity recognition.

Federated learning presents another avenue for future research in HAR, with potential benefits in both performance improvement and privacy preservation. By enabling data sharing across multiple devices, federated learning allows HAR systems to learn from diverse, real-world data without directly accessing users’ sensitive information. This approach can lead to more robust models that can better generalize to different populations and contexts while respecting users’ privacy. Researchers should focus on optimizing federated learning algorithms, as well as addressing challenges related to communication efficiency, data heterogeneity, and security in distributed learning settings.

Another vital aspect of HAR in smart living services and applications is human-centered design. A multidisciplinary approach that involves collaboration between computer scientists, engineers, psychologists, and social scientists is essential for ensuring that HAR systems meet the diverse needs and preferences of end-users. By prioritizing user experience and incorporating insights from various fields, researchers can develop more intuitive, adaptable, and user-friendly HAR systems that seamlessly integrate into people’s everyday lives. Future research should emphasize the importance of human-centered design principles, investigate novel interaction modalities, and explore methods for eliciting user feedback and preferences to inform system development.

The importance of overall system design, particularly emphasizing low-power consumption and lightweight processing, must be considered for smart living services and applications. Despite this, many studies still need adequate attention to these crucial aspects. As smart environments frequently face limitations in energy consumption, device size, and battery life, developing energy-efficient and lightweight solutions becomes imperative. Energy-harvesting wearable devices, which can capture and store energy from various sources like solar, thermal, or kinetic energy, can significantly mitigate energy consumption concerns. Employing such energy-harvesting methods makes it possible to extend the battery life of wearable devices or even eliminate the need for batteries, substantially reducing the system’s overall energy consumption. Additionally, low-power ML algorithms for HAR can help minimize energy usage without compromising performance. These algorithms can be designed to run on resource-constrained devices, such as microcontrollers or edge devices, enabling HAR to be processed locally. This reduces the need for transmitting data to the cloud, which can be power-intensive, and results in lower latency and increased privacy. To further enhance the energy efficiency of smart living services and applications, it is important to optimize both hardware and software components. This optimization could involve employing energy-efficient processors, memory, and communication modules on the hardware side. On the software side, researchers can focus on developing lightweight algorithms that require minimal computational resources and can adapt dynamically to the available energy budget. Smart living services and applications can become more viable and sustainable in the long run by prioritizing low-power consumption and lightweight processing in the overall system design.

Multi-resident HAR represents an important area for further exploration, as most existing studies concentrate on single-occupant scenarios. The ability to accurately detect and analyze the actions of multiple individuals in a shared environment opens up many practical applications, addressing diverse needs across various sectors. In assisted living facilities, for instance, multi-resident HAR can significantly enhance residents’ quality of care and support. By simultaneously monitoring the activities of multiple individuals, caregivers can receive real-time updates on each resident’s well-being, enabling timely interventions if necessary. It is particularly beneficial for detecting falls, wandering, or other behaviors requiring immediate attention, ultimately contributing to a safer and more responsive living environment. Smart homes also stand to benefit greatly from advancements in multi-resident HAR. By recognizing the activities of various family members, smart home systems can make personalized environmental adjustments, such as controlling lighting, temperature, and entertainment settings based on individual preferences and habits. Additionally, multi-resident HAR can bolster security measures by identifying and differentiating between authorized family members and potential intruders. Addressing the challenges associated with multi-resident HAR will likely involve refining existing techniques and developing novel approaches. For example, researchers may need to devise innovative ways to differentiate between the actions of multiple individuals, even when their activities overlap or occur nearby. Furthermore, integrating data from various sensor types, including wearable devices, cameras, and ambient sensors, could enhance the accuracy and reliability of multi-resident HAR systems.

Lastly, it is essential to address ethical considerations and privacy concerns in smart living environments that employ HAR systems. While recent advances in privacy-preserving techniques have made some progress, privacy remains a significant concern in HAR. Researchers should continue exploring ways to develop secure and privacy-preserving HAR systems that protect individuals’ data and privacy, such as through differential privacy, homomorphic encryption, or secure multi-party computation. In addition, the vulnerability of HAR models to adversarial attacks and the connection between adversarial optimization and interpretability warrant further investigation. Developing explainable HAR models that provide transparent and interpretable insights into their decision-making processes can help build trust and facilitate user acceptance of these systems in smart living services and applications.

In light of the above discussions on open issues and future research directions in HAR for smart living, several actionable insights and recommendations can be highlighted:

- Integration of sensor fusion techniques: Exploring the integration of multiple sensing technologies, such as wearable devices, cameras, and ambient sensors, can significantly enhance human activity recognition (HAR) systems in smart living. By combining data from different sensors, a more comprehensive understanding of users’ activities can be achieved, leading to improved accuracy and reliability in activity recognition.
- Investigation of federated learning for HAR: Further research should be conducted to explore the potential benefits of federated learning in HAR systems. This approach allows HAR models to learn from diverse real-world data while preserving user privacy by enabling data sharing across multiple devices. Optimizing federated learning algorithms and addressing challenges related to communication efficiency, data heterogeneity, and security can result in more robust models that generalize well to different populations and contexts.
- Adoption of human-centered design principles: Incorporating human-centered design principles in the development of HAR systems is essential. Collaboration among experts from various disciplines can lead to the creation of intuitive and user-friendly systems that meet the diverse needs and preferences of users. Exploring novel interaction modalities and incorporating user feedback can enhance the usability and adaptability of HAR systems in smart living environments.
- Emphasis on low-power consumption and lightweight processing: Prioritizing energy-efficient and lightweight solutions is crucial for HAR systems in smart living. Exploring energy-harvesting wearable devices, optimizing hardware and software components for low-power consumption, and developing efficient machine learning algorithms can minimize energy usage and enable local processing, resulting in longer battery life, reduced latency, and increased privacy.
- Exploration of multi-resident HAR: There is a need to investigate the accurate detection and analysis of activities from multiple individuals in shared living environments. Advancing multi-resident HAR can enhance the quality of care in assisted living facilities and enable personalized adjustments in smart homes. Addressing challenges related to differentiating between multiple individuals’ actions and integrating data from various sensor types can contribute to the development of more comprehensive and effective multi-resident HAR systems.
- Addressing ethical and privacy concerns: Ensuring the development of secure and privacy-preserving HAR systems is of utmost importance. Exploring techniques such as differential privacy, homomorphic encryption, and secure multi-party computation can protect individuals’ data and privacy. Additionally, developing explainable HAR models and investigating adversarial attacks can enhance system transparency, trust, and user acceptance in smart living applications.

One potential limitation of this study is the limited exploration of the interplay and integration of the dimensions of HAR in smart living with other key aspects of the smart living ecosystem. While the study focuses on four important dimensions, namely context awareness, data availability, personalization, and privacy, it does not extensively examine how these dimensions interact and integrate with other dimensions within the broader smart living framework.

## 8. Conclusions

This comprehensive review has meticulously examined the role of HAR within the realm of smart living, delving into its various dimensions and pinpointing both the challenges and opportunities that lie ahead for future research. The proposed framework emphasizes the critical importance of context awareness, data availability, personalization, and privacy, in the context of smart living services and applications. Through a critical analysis of these aspects, this review accentuates the necessity to tackle biases and inaccuracies, manage the complexity and privacy concerns, strike a balance between real-time processing and resource efficiency, and prioritize privacy-preserving techniques. The comparative advantages lie in its comprehensive coverage of the dimensions crucial for smart living, addressing the limitations of previous reviews, and providing a solid foundation for further advancements in the field.

As we look to the future, researchers should concentrate on refining and amalgamating data availability approaches, devising innovative synthetic data generation techniques, optimizing federated learning algorithms, and delving into the individual sensing technologies and systemic aspects of HAR systems. In addition to these technical advancements, addressing the challenges of accuracy, reliability, scalability, and adaptability in smart living services and applications is of paramount importance for the development of effective, secure, and ethical HAR solutions. Prioritizing low-power consumption and lightweight processing in system design, researchers can contribute to the creation of more sustainable, accessible, and efficient smart living solutions that cater to a wide range of users and environments. This will, in turn, enhance the quality of life for those who reside in smart living spaces, promoting a more comfortable, safe, and convenient living experience.

The development of multi-resident HAR represents a crucial area for further exploration, as it has significant practical applications in assisted living facilities and smart homes. The ability to recognize and interpret the activities of multiple individuals simultaneously can contribute to a safer, more responsive, and personalized living environment. For instance, in an assisted living facility, multi-resident HAR systems can monitor the well-being of the elderly and provide timely assistance when required, ensuring their safety and independence. Similarly, in a smart home setting, these systems can facilitate energy conservation, enhance security, and enable seamless interaction between the residents and their environment.

Moreover, addressing the ethical implications of HAR systems is essential, as the widespread adoption of these technologies raises concerns regarding user privacy, data ownership, and potential misuse of sensitive information. Researchers should work towards establishing clear ethical guidelines and developing privacy-preserving techniques that protect user data while still enabling effective HAR solutions. In light of the rapid advancements in AI, ML, and sensor technologies, the potential of HAR systems in smart living services and applications is immense. However, realizing this potential requires a multidisciplinary approach, bringing together researchers from various fields such as computer science, engineering, psychology, and social sciences. This collaboration will help bridge the gap between technology and human-centered design, ensuring that HAR systems not only meet technical requirements but also address the diverse needs and preferences of the end-users.

Ultimately, by overcoming the challenges and leveraging the opportunities highlighted in this review, researchers and practitioners can develop innovative, robust, and user-friendly HAR systems that seamlessly integrate into smart living spaces, transforming the way we live and interact with our environment.

In the continuum of our review study, our ongoing and future work will concentrate on a broader exploration of how HAR intertwines with multifarious facets of smart living. Our approach entails a comprehensive examination of empirical research and real-world applications that incorporate HAR into diverse areas of the smart living ecosystem. We aim to uncover potential synergies, dependencies, and trade-offs that coexist between HAR and these varying dimensions of smart living. To this end, our study will encourage and incorporate interdisciplinary research collaborations to facilitate an exhaustive investigation into the abundant scholarly works trailing overlapping domains. The goal is to condense and amalgamate information on established methodologies, frameworks, and standards that pave the way for the effortless integration of HAR into a wide array of smart living services and applications.

## Figures and Tables

**Table 1:** Common Publicly Available Datasets.

Reference	Name	Sensors	Subjects No/Type of Environment	Actions/Contexts
Roggen et al. [44]	Opportunity	Getting up, grooming, relaxing, preparing and consuming coffee and a sandwich, and cleaning up; opening and closing doors, drawers, fridge, dishwasher, turning lights on and off, and drinking in different positions.	12	Body-worn, object-attached, ambient sensors (microphones, cameras, pressure sensors).
Reiss and Stricker [45]	PAMAP2	Lie, sit, stand, walk, run, cycle, Nordic walk, iron, vacuum clean, rope jump, ascend and descend stairs, watch TV, computer work, drive car, fold laundry, clean house, and play soccer.	9	Inertial Measuring Units (IMUs), ECG.
Cook and Diane [46]	CASAS: Aruba	Movement from bed to bathroom, eating, getting home, housework, leaving home, preparing food, relaxing, sleeping, washing dishes and working.	1 adult, 2 occasional visitors	Environment sensors: motion, light, door and temperature.
Cook et al. [47]	CASAS: Cairo	Bed (four different types), bed to toilet, breakfast, dinner, laundry, leave home, lunch, night wandering, resident1 work, resident2 medicine.	2 adults, 1 dog	Environment sensors: motion and light sensors.
Cook and Schmitter-Edgecombe [48]	CASAS: Kyoto Daily life	Making a call, washing hands, cooking, eating and washing the dishes.	20	Environment sensors: motion, associated with objects, from the medicine box, a flowerpot, a diary, a closet, water, kitchen and telephone use sensors.
Cook et al. [50]	CASAS: Tokyo	Working, preparing meals, and sleeping.	2	Environment sensors: motion, door closure, light.
Weiss et al. [51]	WISDM	Walking, jogging, stairs, sitting, standing, kicking a soccer ball, dribbling a basketball, catch with a tennis ball, typing, writing, clapping, brushing teeth, folding clothes, eating (pasta, soup, sandwich, chips), and drinking from a cup.	51 (undergraduate and graduate university students between the ages of 18 and 25)	Accelerometer and gyroscope sensors, which are available in both smartphones and smartwatches.
Singla et al. [49]	CASAS: Kyoto Multiresident	Fill medication dispenser, hang up clothes, move couch and coffee table, sit on couch, water plants, sweep kitchen floor, play checkers, set out dinner ingredients, set dining room table, read magazine, simulate electric bill payment, gather picnic food, retrieve dishes from cabinet, pack supplies in picnic basket, pack food in picnic basket.	2 (pairs taken from 40 participants)	Environment sensors: motion, item, cabinet, water, burner, phone and temperature.
Cook and Schmitter-Edgecombe [48]	CASAS: Milan	Bathing, bed to toilet, cook, eat, leave home, read, watch TV, sleep, take medicine, work (desk, chores), meditation.	1 woman, 1 dog, 1 occasional visitor	Environment sensors: motion, temperature, door closure.
Vaizman et al. [52]	ExtraSensory	Sitting, walking, lying, standing, bicycling, running outdoors with friends, talking with friends, exercise at the gym, drinking, sitting at home watching TV, traveling on a bus while standing.	60	Accelerometers, gyroscopes, and magnetometers sensors, which are available in both smartphones and smartwatches.
Banos et al. [53]	MHEALTH	Standing still, sitting and relaxing, lying down, walking, climbing stairs, waist bending forward, frontal elevation of arms, knees bending (crouching), cycling, jogging, running, jump front & back	10	Accelerometers, gyroscopes, magnetometers, EEG.
Soomro et al. [54]	UCF101	N. 101 action classes divided into five types: Human-Object Interaction, Body-Motion Only, Human-Human Interaction, Playing Musical Instruments, Sports.	N.A.	RGB video clips (25 FPS, 320 × 240 pixels).
Kuehne et al. [55]	HMDB51	N. 51 action categories grouped into five types: general facial actions, facial actions with object manipulation, general body movements, body movements with object interaction, and body movements for human interaction.	N.A.	RGB video clips.
Shahroudy et al. [56]	NTU RGB+D	N. 40 daily actions (e.g., drinking, eating, reading), 9 health-related actions (e.g., sneezing, staggering, falling down), and 11 mutual actions (e.g., punching, kicking, hugging).	40	3 Microsoft Kinect v2 sensors located at the same height but from three different horizontal angles: −45°, 0°, and +45°.
Riboni et al. [57]	SmartFABER	Preparing food, consuming meal, taking medicines, opening and closing of drawers, fridge and cabinet doors, use of appliances, non-critical anomalies, critical anomalies.	21 elderly individuals in a smart home laboratory (7 healthy seniors, and 14 with early symptoms of MCI).	Presence, contact, pressure, RFID, magnetic.
Climent-Perez et al. [58]	PAAL ADL Accelerometry	Six broad categories: eating and drinking, hygiene/grooming, dressing and undressing, miscellaneous and communication, basic health indicators, and house cleaning.	52 (26 women, 26 men)	Wrist-worn device with accelerometer.
van Kasteren et al. [59]	Houses: HA, HB, and HC.	Sleeping, leaving the house, toileting, showering, having breakfast, having dinner, and drinking	1	Reed switches, pressure mats, mercury contacts, passive infrared (PIR), float sensors.
Anguita et al. [60]	UCI-HAR	Standing, sitting, laying down, walking, walking downstairs, and walking upstairs.	30	Accelerometers and gyroscopes embedded in a Samsung Galaxy S II smartphone.
Ordonez et al. [61]	Ordonez	Leaving, toileting, showering, sleeping, breakfast, dinner, drink, idle/unlabeled, lunch, snack, spare time/tv, grooming.	N.A.	PIR sensors (motion detection), reed switches (open/close states of doors and cupboards), float sensors (flushing of toilets).
Shoaib et al. [62]	Utwente	Walking, jogging, biking, walking upstairs, walking downstairs, sitting, standing, eating, typing, writing, drinking coffee, giving a talk, smoking.	10	Accelerometer, a gyroscope, and linear acceleration sensors located on the wrist and in the pocket.
Imran et al. [63]	IITR-IAR	Clapping, crouching, hopping, running, walking, waving, dropping object, carrying/pointing a gun, picking up object, recording video, clicking selfie, throwing object, chasing, fighting, handshaking, hugging, kicking, passing object, punching, pushing.	35	Cameras.

**Table 2:** Context awareness.

Reference	Methods	Dataset/s	Performance	Sensor/s	Actions
Xu et al. [64]	Marker-based Stigmergy, DwN.	CASAS (Aruba)	R = 0.9669, P = 0.9598, F1S = 0.9633.	Environment sensors (CASAS).	Cooking, watching TV, reading, and sleeping
Li et al. [65]	Spatial Distance Matrix, Contribution Significance Analysis, Time-Domain CNN.	CASAS (Cairo, Milan, Kyoto)	A = 0.9708, P = 0.9535, R = 0.9611, F1S = 0.9571.	Environment sensors (CASAS).	All activities included in Cairo, Milan, and Kioto datasets.
Kim [66]	Activity Recognition Transformer.	CASAS, Self-collected	A = 0.955, P = 0.962, R = 0.955, F1S = 0.954.	Environment sensors (CASAS), brightness, speaker recognition, sound level, light use, person presence, seat occupying.	Chatting, seminar, technical discussion, and group study in the seminar room testbed, and move furniture, play a game, prepare for dinner, and pack a picnic.
Ehatisham-ul-Haq et al. [67]	Supervised Machine Learning, Boosted Decision Tree (DT), Neural Network Classifiers.	ExtraSensory	A = 0.8943 (avg.).%.	Smartphone and smartwatch accelerometers	Sitting, walking, lying, standing, running, and bicycling.
Buoncompagni et al. [68]	Ontology networks, logic-based reasoning.	CASAS	F1S = 0.78 (min.), F1S = 0.98 (max).	Refer to CASAS (no further details provided).	Refer to CASAS (no further details provided).
Javed et al. [69]	Deep recurrent neural network (DRNN), Recurrent neural networks (RNNs), Smart city, Internet of things (IoT).	Self-collected involving 12 subjects.	A = 0.9943 (max.).	Accelerometer, gyroscope, magnetometer in smartphone and Google Fit.	In a vehicle, on foot, still, tilting, walking.
Ehatisham et al. [70]	Decision Tree (DT), Random Forest (RF), and Neural Networks (NN).	ExtraSensory.	A = 0.83	Accelerometers, gyroscopes.	All activities of ExtraSensory datasets.
Ceron et al. [71]	K-Nearest Neighbor (KNN), Naive Bayes (NB), and Hoeffding Tree (HT).	Self-collected, 22 participants (11 young people, and 11 older adults)	F1 = 0.88	IMU placed in the participants’s shoe, and Bluetooth low energy beacons (BLE) deployed in the indoor environment.	Walking, Climbing, Being still, Using jug, Sweeping, Using Bathroom sink, Using toilet.
Srihari et al. [72]	Deep-learning-based Spatio-temporal recognition, frame-based ROI detection.	IITR-IAR.	A = 0.985 (avg.).	FLIR T1020 camera, FLIR ONE thermal camera.	All activities of IITR-IAR dataset.
Mohamed et al. [73]	Adaptive Profiling Model using multi-label classification, Label Combination(LC)-Random Forest (RF).	CASAS.	A = 0.99 (avg.)	Ambient sensor data of the CASAS datasets.	Medication dispenser, reading magazine, sweeping floor, setting table for dinner, reading magazine, gathering picnic food, retrieving dishes from cabinet, packing supplies for picnic food, hanging clothes, move furniture, watering plants, playing checkers, prepare dinner, pay bills, retrieving dishes from cabinet, packing picnic food.
Sridharan et al. [74]	Sequence matching, DTW algorithm.	Self-collected.	A = 0.918 (avg.).	Low power transmitting wearable beacon with embedded sensors clipped on to the shirt collar.	Five micro-activities: Sitting on Centre of Couch, Sitting on Left of Couch, Sitting on Right of Couch, Using the Shower, Using the Bathroom Sink. Walking routes: Bathroom to Kitchen Fridge, Kitchen Fridge to Bathroom, Kitchen Fridge to Sink, Couch to Front Door, Couch No.2 to Front Door.

**Table 3:** Data Availability.

Reference	Methods	Dataset/s	Performance	Sensor/s	Actions
Vishwakarma et al. [75]	Human micro-Doppler signatures, motion capture, CLEAN algorithm, spectrograms.	Synthetically generated.	A = 0.694 (min.), A = 0.9784 (max.).	WiFi (simulated), Kinect (motion capture).	Rotating body, kicking, punching, grabbing an object, walking back/forth in front of the radar, standing up from a chair, sitting down on a chair, human walk to fall, standing up from the ground to walk.
Riboni et al. [77]	Hidden Markov Models (HMMs), Viterbi Algorithm, OWL 2 Ontology.	CASAS	A = 0.7213.	Passive Infrared Motion Sensors, Temperature Sensors, Door Sensors, Furniture Sensors, Item Sensors.	Fill a medication dispenser, hang up clothes, move the couch and coffe table, sit on the couch and read, water plants, sweep the kitchen floor, play a game of checkers, set out ingredient for dinner, set dining room table, pay an electric bill, prepare a picnic basket, retrieve dishes, pack supplies in the picnic basket.
Dhekane et al. [78]	Similarity-based Change Point Detection (S-CPD), Sensor Distance Error (SDE), Feature Extraction, Classification, Noise Handling, Annotations.	CASAS (Aruba, Kyoto, Tulum, and Milan).	A = 0.9534 (min.), A = 0.9846 (max.).	Motion, light, door and temperature, associated with objects.	All activities included in Aruba, Kyoto, Tulum, and Milan.
Zilelioglu et al. [81]	Semi-supervised generative adversarial networks (GANs) using temporal convolutions.	PAMAP2, Opportunity.	A = 0.90.	Wearable IMUs, objectsequipped with sensors, and ambient sensors.	All activities of PAMAP2, Opportunity-locomotion, and LISSI HAR dataset.
Nan et al. [76]	Graph Convolutional Networks (GCN), Temporal Convolutional Networks (TCN).	NTU RGB+D	A = 0.8273 (min.), A = 0.9825 (max.).	Microsoft Kinect v2 sensors.	Drinking, eating, reading, writing, brushing teeth, sneeze/cough, staggering, falling, touch head (headache), touch chest (stomachache/heart pain), touch back (backache), touch neck (neckache), nausea or vomiting condition, use a fan (with hand or paper)/feeling warm, punching/slapping another person, kicking another person, pushing another person, etc.
Civitarese et al. [79]	OWL 2 ontology, Markov Logic Network (MLN), Hidden Markov Model (HMM), probabilistic and ontological reasoning, semantic correlations, temporal reasoning.	CASAS, SmartFABER.	A = 0.61 (min.), A = 0.80 (max.), F1S = 0.67 (min.), F1S = 0.76 (max).	Presence, contact, pressure, RFID, magnetic, motion, light, door, temperature.	Fill medication dispenser, watch DVD, water plants, answer the phone, prepare birthday card, prepare soup, clean, choose outfit, taking medicines, cooking, eating.
Liaqat et al. [80]	Random forest, KNN, logistic regression (LR), multilayer perceptron (MLP), decision tree, quadratic discriminant analysis (QDA), SVM, CNN, and long short-term memory (LSTM).	Self-collected involving 30 subjects.	A = 0.98 (max).	Accelerometer, gyroscope, and magnetometer in the smartphone.	Standing, sitting, laying, walking, walking downstairs and walking upstairs.

**Table 4:** Personalization.

Reference	Methods	Dataset/s	Performance	Sensor/s	Actions
Gholamiangonabadi et al. [82]	Stationary Wavelet Transform, Empirical Mode Decomposition (EMD), Ensemble EMD.	MHEALTH, WISDM.	A = 0.912 (avg., MHEALTH), A = 0.576 (avg., WISDM).	Accelerometers, gyroscopes, magnetometers.	All activities of MHEALTH and WISDM datasets.
Kwon et al. [84]	Personalized anomaly detection criteria, MMSE score, Shapiro–Wilk test, Wilcoxon rank-sum test, Spearman correlation analysis, random forest.	Self-collected, 13 participants (7 healthy seniors, 6 early-stage dementia).	A = 0.912	Environmental sensors (installed on household appliances and various locations): door sensors, motion sensors, temperature-humidity sensors, vibration sensors, lidar sensors, and smart plugs.	Using the telephone, shopping, preparing food/cooking, household chores, using transportation, walking outdoors, taking medications, managing finances, grooming, using household appliances.
Hamad et al. [85]	Dilated causal convolution, multi-head self-attention mechanisms.	Houses, Ordonez, UCI-HAR.	F1S = 0.7393 (min.), F1S = 0.9224 (max.).	Embedded binary sensors, inertial wearable sensors.	All activities from used datasets (see Houses, Ordonez, and UCI-HAR datasets).
Gorjani et al. [86]	Multilayer perceptron (MLP) neural network.	Self-collected.	A = 0.98 (avg.).	Two individual wearable gadgets based on STMicroelectronics development boards with 3-axis magnetometer, 3D accelerometer, and 3D gyroscope worn on wrist- and ankle-worn.	Climbing down the stairs, Climbing up the stairs, Using a computer, Relaxing, Running, Standing, Vacuum cleaning, Walking, Writing using a pen.
Mekruksavanich et al. [87]	Gate recurrent unit (GRU), bidirectional GRU (BiGRU), CNN BiGRU, LSTMs, BiLSTMs.	Utwente, PAMAP2, WISDM.	A = 0.8209 (min), A = 0.9878 (max.), P = 0.8625 (min), P = 1.0000 (max), R = 0.9110 (min.), R = 0.9889 (max), F1S = 0.8561 (min.), F1s = 1.0000 (max.).	Accelerometer, magnetometer, and gyroscope in two smartphones worn in right pants pockets and on right wrists (emulating a smartwatch).	Walking, standing, jogging, sitting, biking, walking upstairs/downstairs, typing, writing, drinking, talking, smoking, eating, lying, running, cycling, vacuum cleaning, ironing, brushing teeth.
Sanabria et al. [88]	Ensemble learning, Variational autoencoder, feature alignment.	Houses (HA, HB, HC), CASAS (Aruba, Twor).	F1S = 0.547 (min.), F1S = 0.917 (max.).	Wireless motion sensor, passive infrared (PIR), switch, pressure sensors.	Leaving house, toileting, showering, having breakfast, having dinner, and drinking, meal preparation, eating, working, sleeping, bed to toilet transition, housekeeping.
Ganesh et al. [90]	Random Forest Classifier.	Self-collected, 4 male subjects.	A = 0.989.	RGB camera.	Gym activities:push-up, squat, plank, forward lunge, and sit-up.

**Table 5:** Privacy.

Reference	Methods	Dataset/s	Performance	Sensor/s	Actions
Shen et al. [99]	Federated Meta-Learning, CNN-based attention module, cluster-specific features.	Two self-collected datasets with 30 and 48 participants.	A = 0.8395 (min.), A = 0.9348 (max.), F1S = 0.7836 (min.), F1S = 0.9037 (max.).	Motion-reactive sensors such as accelerometer, gyroscope, linear acceleration, gravity, rotation vector, and magnetic field sensors, as well as sensors for location, phone state, temperature, atmospheric pressure, humidity, proximity, WIFI network, running application, screen status, flight mode, battery charge, battery level, doze modality, headset plugged in, audio mode, music playback, audio from the internal mic, notifications received, touch event, and cellular network info.	Housework, self-care, eating, study, lesson, social life, watching TV shows or movies, social media usage, traveling, coffee break, phone calling or chat, reading or listening, hobbies, work, and rest/nap.
Yin et al. [98]	Butterworth filter, LSTM	Self-collected involving one subject.	A = 0.98287 (avg).	Low-resolution (8x8) infrared array.	Lying, standing, sitting, walking, and empty.
Iravantchi et al. [95]	Raspberry Pi, infrasound frequencies, Fast Fourier Transform, Principal Component Analysis, Random Forest Classifer.	Self-collected in three homes and four commercial buildings.	A = 0.914 (avg).	Microphones	127 everyday household and workplace objects.
Climent-Pérez et al. [104]	Many-objective evolutionary algorithm.	PAAL ADL Accelerometry.	A = 0.68	Wrist-worn devices equipped with accelerometers.	All activities of PAAL v2.0 dataset
Zhang et al. [105]	CNN.	Self-collected.	A = 0.90.	Off-the-shelf FMCW radar operating at C-band (5.8 GHz).	Walking, sitting down, standing up, picking up an object, drinking water, and falling.
Beaulieu et al. [106]	Deep learning model combining EfficientNetB0 and LSTM neural networks using transfer learning and minimalist data pre-processing.	Self-collected, 10 participants.	A = 0.655.	Three XeThru X4M200 Ultra-Wideband (UWB) radars.	Drinking, Sleeping, Putting on Jacket, Cleaning, Cooking, Making Tea, Doing the Dishes, Brushing teeth, Washing hands, Reading, Eating, Walking, Putting on Shoes, Taking Medication, Using Computer.
Shang et al. [107]	LSTM-CNN.	Self-collected, 5 participants in a classroom.	A = 0.941.	WiFi signal transmitter and a Channel State Information (CSI) receiver.	Two static movements of standing and sitting, and three dynamic movements of falling, standing up and stepping.
Yan et al. [97]	Inflated 3D ConvNet, Mask-Residual Convolutional Network (RCN).	UCF101, HMDB51.	A = 0.611 (min.), A = 0.931 (max.).	RGB camera	All activities included in UCF101 and HMDB51 datasets.
Arrotta et al. [102]	Explainable AI, Grad-CAM, LIME, Model Prototypes, CNNs.	Self-collected, CASAS.	F1S = 0.90 (avg., Self-collected), F1S = 0.80 (avg., CASAS)	Magnetic sensors (doors and drawers), pressure mats, smart-plugs, and inertial sensor in smartwatches.	Answering phone, clearing table, cooking a hot meal, eating, entering home, leaving home, making a phone call, cooking a cold meal, setting up table, taking medicines, working, washing dishes, watching TV.
Yu et al. [103]	Federated learning, semi-supervised online learning.	Self-collected involving 15 subjects, UCI-HAR.	A = 0.8169 (avg., Self-collected), A = 0.9268 avg., (HAR-UCI), F1S = 0.7998 (avg., Self-collected) F1S = 0.9232 (avg., UCI-HAR).	Self-collected: accelerometer, gyroscope, and magnetometer on 7 body parts, including chest, one forearm, head, shin, one thigh, one upper arm and waist; UCI-HAR: accelerometer and gyroscope, 3-axial linear acceleration and 3-axial angular velocity of a smartphone on the waist.	Running, standing, lying, sitting, walking, jumping, climbing stairs down and up, walking upstairs, downstairs, sitting, standing, laying.

**Table 6:** Smart Living Services and Applications.

Reference	Type	Description	Methods and Techniques
[108]	Assisted Living	HAR system for assisted living, designed to monitor the vital signs and home automation of patients in order to reduce pressure on the social health insurance system.	Object detection, Neural network, Human–Object Interaction (HOI) detection, Scene understanding, NVIDIA Jetson AGX processing unit, CNNs, MQTT Protocol.
[109]	Assisted Living	Assist in monitoring the well-being of elderly, and can be used in situations like the COVID-19 pandemic to remotely monitor patients.	Segmentation (activity, sensor, time, area), Features (Time Domain, Frequency Domain Environment), Supervised Learning, K-Nearest Neighbor (KNN), Random Forest Classifier (RFC), Decision Tree (DT), Naïve Bays (NB), Linear Support Vector Machine (SVM), Ensemble Model.
[110]	Assisted Living	HAR for elderly people in smart homes.	Naive Bayes supervised learning algorithm, Prediction model for ADL, SVM, Linear Regression (LR), and K-Nearest neighbors (K-NN), CASAS dataset.
[19]	Assisted Living	The system monitors and assesses the health of the elderly and also records their action histories and behaviors, reducing the workload of caregivers as an ambient assisted living system.	Stereo depth camera, UV-disparity maps, Spatial-temporal features, Depth motion appearance (DMA), Depth motion history (DMH), Histogram of Oriented Gradients (HOG) descriptor, Automatic rounding method, Continuous long frame sequences
[122]	Assisted Living	The system identifies behavioral patterns and detects anomalies in the activities of older persons through ADL applications and IoT data	Large-scale sensor data, Anomaly detection, Parametric statistical approach, Self-reported routines, Internet of things (IoT) devices, Real-time monitoring, SMS-based notification service, Off-the-shelf sensors, Uncontrolled environment.
[123]	Assisted Living	Classification scheme for fall detection and prevention in smart home AAL.	Argumentation enabled devices, Fuzzy argument based classification scheme (CleFAR), Fall Activity Recognition (FAR), Fall prevention system, Random Forest (RF), SVM, Naive Bayes (NB), Decision Tree (DT), Artificial Neural Networks (ANN), Weighted Voting Scheme (WVS), Wearable fall detection systems.
[124]	Assisted Living	Complex human activities prediction from a single accelerometer sensor using a local weighted machine learning approach.	Locally Weighted Random Forest (LWRF) machine learning algorithm, Time and frequency features, PAAL ADL Accelerometry Dataset, Gender recognition, Accelerometer signal domain, Mental status tracking.
[111]	Health Status Surveillance	Non-intrusive monitoring wellbeing of dementia patients living alone using smart meter load disaggregation.	SVM classifier, Random Decision Forest (RDF) classifier.
[112]	Health Status Surveillance	Multi-dish food recognition model to improve dietary intake reporting in the context of preventive healthcare.	EfficientDet-D1, EfficientNet-B1, bidirectional feature pyramid network (BiFPN). Comparison with: SSD Inception V2, Faster R-CNN Inception ResNet V2.
[113]	Health Status Surveillance	Monitoring of physical activities of elderly people using smartphone.	Deep learning models, smartphone accelerometer sensor data, UCI and WISDM datasets.
[125]	Health Status Surveillance	Context-awareness system for human-robot scene interpretation in ambient assisted living scenarios, particularly for the elderly, improving robot performance and activity recognition.	Topological Bayesian network (BN) models, learning and inferring informal relationships, OpenMarkov.
[126]	Health Status Surveillance	Monitoring activities of daily living (ADLs) and detecting abnormalities in occupant behavior.	Fuzzy Ontology Activity Recognition (FOAR), fuzzy temporal ontologies, Fuzzy Semantic Web Rule Language (SWRL).
[114]	Health Hazard Surveillance	Highly accurate bathroom activity recognition system using privacy-preserving infrared proximity sensors.	Raspberry Pi devices, Wi-Fi, Bluetooth, Bluetooth Low Energy, WebSockets for real-time data transfers.
[115]	Health Hazard Surveillance	Recognize normal activities of elderly residents, separate them from anomalous activities, and identify anomalous days based on the number of activities performed in a day.	Probabilistic Neural Network (PNN), H2O autoencoder for anomaly detection, curve fitting (variations from the mean in daily activities).
[116]	Energy Management	Save energy by dynamically changing the setpoint of a connected thermostat through human activity recognition based on computer vision while preserving occupant’s thermal comfort.	RGB-Depth cameras, skeleton-based models over 3D representation, Recurrent Neural Networks (RNN) for Human Activity Recognition (HAR), Long Short-Term Memory Networks (LSTMs), and EnergyPlus™ for energy consumption simulations.
[117]	Energy Management	Building Energy and Comfort Management (BECM) system that monitors, recognizes, and predicts user preferences and habits related to appliance usage.	Probabilistic Prediction, Scheduling Algorithm.
[118]	Security Surveillance	Multimodal approach for recognizing suspicious human activities in smart city security using computer vision and Internet of Things (IoT) technology.	YOLO-v4, 3D-CNN, intersection over union (IOU), Internet of Things (IoT)-based architecture, UCF-Crime and MS-COCO datasets.
[119]	Security Surveillance	Classify children’s activities (running, playing, crying, and walking) using environmental sound.	Audio recordings from smartphones, time-domain and frequency-domain features, Python programming language, PyAudio-Analysis library, and SVM algorithm.
[120]	Natural Interaction	Classify human gross-motor activities and arm gestures based on phase information from high-resolution radar range maps.	Histogram of Oriented Gradients (HOG) for feature extraction, Nearest Neighbor (NN), linear SVM, Gaussian SVM for classification, and feature fusion of different data domains.
[121]	Natural Interaction	Human activity and gesture recognition schemes using CSI provided by WiFi devices.	Hampel identifier algorithm for preprocessing, RGB image creation from CSI data, data augmentation to reduce overfitting, Deep CNNs (AlexNet, VGG19, and SqueezeNet) for classification and feature extraction.

## Data Availability

Not applicable.

## Abbreviations

The following abbreviations are used in this manuscript:

AAL	Ambient Assisted Living
ADL	Activities of Daily Living
AI	Artificial Intelligence
BiGRU	Bi-directional Gated Recurrent Unit
CNN	Convolutional Neural Network
CPD	Change Point Detection
CSI	Channel State Information
DE	Differential Evolution
DL	Deep Learning
DT	Decision Tree
GRU	Gated Recurrent Unit
HAR	Human Action Recognition
HMM	Hidden Markov Model
IMU	Inertial Measuring Unit
ICT	Information and Communication Technology
IoT	Internet of Things
KNN	K-Nearest Neighbors
LR	Logistic Regression
LSTM	Long Short-Term Memory
LSVM	Linear Support Vector Machine
ML	Machine Learning
MLP	Multilayer Perceptron
PIR	Passive Infrared
RCN	Residual Convolutional Network
RF	Random Forest
RGB	Red-Green-Blue
RNN	Recurrent Neural Network
SDE	Sensor Distance Error
SVM	Support Vector Machine
TCN	Temporal Convolutional Network
UWB	Ultra-Wideband
